# Identification of Senomorphic miRNAs in Embryonic Progenitor and Adult Stem Cell‐Derived Extracellular Vesicles

**DOI:** 10.1111/acel.70071

**Published:** 2025-04-24

**Authors:** Tianpeng Zhang, Allancer D. C. Nunes, Jieun Lee, Dana Larocca, Giovanni Camussi, Sai Kiang Lim, Vicky U. Bascones, Luise Angelini, Ryan D. O'Kelly, Xiao Dong, Laura J. Niedernhofer, Paul D. Robbins

**Affiliations:** ^1^ Masonic Institute on the Biology of Aging and Metabolism University of Minnesota Minneapolis Minnesota USA; ^2^ Department of Biochemistry, Molecular Biology and Biophysics University of Minnesota Minneapolis Minnesota USA; ^3^ AgeX Therapeutics, Inc. Alameda California USA; ^4^ Department of Medical Science University of Torino Turin Italy; ^5^ Institute of Medical Biology ASTAR Singapore Singapore; ^6^ Department of Genetics, Cell Biology and Development University of Minnesota Minneapolis MN USA

**Keywords:** aging, antiaging, cellular senescence, molecular biology of aging, senescence

## Abstract

Extracellular vesicles (EVs) are secreted by most cell types, transmitting crucial signaling molecules like proteins, small RNAs, and DNA. We previously demonstrated that EVs from murine and human mesenchymal stem cells (MSCs) functioned as senomorphics to suppress markers of senescence and the inflammatory senescence‐associated secretory phenotype (SASP) in cell culture and in aged mice. Here we demonstrate that EVs from additional types of human adult stem cells and embryonic progenitor cells have a senomorphic activity. Based on their miRNA profiles showing prevalence in stem cell EVs versus nonstem cell EVs and the number of age‐related genes targeted, we identified eight miRNAs as potential senomorphic miRNAs. Analysis of these miRNAs by transfection into etoposide‐induced senescent IMR90 human fibroblasts revealed that each of the miRNAs alone regulated specific senescence and SASP markers, but none had complete senomorphic activity. Evaluation of ~300 combinations of miRNAs for senotherapeutic activity identified a senomorphic cocktail of miR‐181a‐5p, miR‐92a‐3p, miR‐21‐5p, and miR‐186‐5p that markedly reduced the expression of *p16* ^*INK4a*^, *p21* ^*Cip1*^, *IL‐1β*, and *IL‐6* and the percentage of SA‐ß‐gal‐positive cells. Transcriptome analysis identified multiple pathways affected by the miRNA cocktail, including cellular senescence and inhibition of PCAF and HIPK2 in the p53 signaling pathway. Finally, treatment of aged mice with liposomes containing the four miRNA cocktail suppressed markers of senescence and inflammation in multiple tissues. These studies suggest that EVs derived from stem cells suppress senescence and inflammation, at least in part, through miRNAs and that a senomorphic miRNA cocktail could be used to target senescence and inflammation to extend health span.

## Graphical abstract

A senomorphic miRNA cocktail (E5), consisting of miR‐181a‐5p, miR‐92a‐3p, miR‐21‐5p, and miR‐186‐5p, was identified from a pool of miRNAs enriched in extracellular vesicles (EVs) derived from multiple functional human stem cell types. E5 reduces senescence and SASP markers both in cell culture and in aged mice. Mechanistically, E5 targets key regulators of the p53 pathway, including PCAF and HIPK2, leading to decreased p53 phosphorylation and acetylation. These effects result in downregulation of *p16^INK4a^*, *p21^Cip1^*, *IL‐1β*, and *IL‐6* expression, and a reduction in SA‐β‐gal+ and γ‐H2AX+ cells across multiple tissues, indicating the senomorphic potential of E5 for healthspan extension.

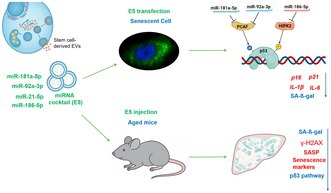

## 1 Introduction

Aging is an important risk factor for most common human diseases, including cancer and fibrotic and neurodegenerative diseases as well as other age‐related diseases (Kennedy et al. 2014). Several common aging mechanisms, termed hallmarks or pillars of aging, that influence lifespan and health span have been identified based on studies across a range of species (Lopez‐Otin et al. 2023, 2013). Of these hallmarks, cellular senescence has received considerable attention recently as a key driver of aging, given it is a druggable target to prevent or treat multiple aging comorbidities and extend health span (Gorgoulis et al. 2019; Zhang et al. 2022).

Cellular senescence is a cell fate that typically includes irreversible loss of proliferative potential, resistance to cell death, increased metabolic activity, and a senescence‐associated secretory phenotype (SASP) of proinflammatory cytokines and chemokines, tissue‐damaging proteases, factors that can impact stem and progenitor cell function, hemostatic factors, and growth factors (Tchkonia et al. 2013). Markers of senescent cells include increases in expression of the cell cycle regulators *p16* ^*INK4A*^ and/or *p21* ^*Cip1*^, SASP factors (e.g., *IL‐6*, *IL‐8*, monocyte chemoattractant protein‐1 [*MCP‐1*], plasminogen‐activated inhibitor‐1 [*PAI‐1*], and many others; Coppe et al. 2010), increased senescence‐associated β‐galactosidase activity (SA‐β‐gal) (Dimri et al. 1995), senescence‐associated distension of satellites (SADS) (De Cecco et al. 2013), and telomere‐associated DNA damage foci (TAFs) (Herbig et al. 2004).

Currently, the two classes of pharmacologic strategies to target senescent cells (SnCs) are senomorphics and senolytics (Zhang et al. 2023). Senomorphics are agents designed to mitigate the effects of senescence usually through the inhibition of the SASP without inducing cell death, whereas senolytics selectively target and eliminate senescent cells. Several senomorphic compounds have been identified, such as rapamycin (Selvarani et al. 2021; Harrison et al. 2009), metformin (Research AFfA 2022; Cabreiro et al. 2013; Anisimov et al. 2008), free radical scavengers (Liu et al. 2024), and inhibitors of IκB kinase (*IKK*) (Tilstra et al. 2012). Examples of senolytics include fisetin (Zhu et al. 2017) the combination of dasatinib and quercetin (Justice et al. 2019; Nambiar et al. 2023) and *Bcl‐2* family inhibitors such as Navitoclax (Liu et al. 2024; Gonzalez‐Gualda et al. 2020).

Stem cell dysfunction is another hallmark of aging, leading to the development of stem cell‐based approaches to extend health span. Currently, numerous ongoing clinical trials use allogenic adult stem cell populations for reducing physical frailty or slowing skin aging (Garay 2023). For example, an allogeneic bone marrow stem cell preparation (Lomecel‐B) has shown positive results in mitigating physical frailty (Golpanian et al. 2016; Yousefi et al. 2022), at least at a specific dose. However, despite their promise, allogeneic stem cell therapies face challenges such as potential immune rejection and the complexity and cost of stem cell isolation, propagation, and transplantation. In addition, the use of autologous stem cells from older donors is limited due to their increased dysfunction with age and disease.

In contrast to allogeneic adult stem cells, extracellular vesicles (EVs) derived from stem cells offer the benefits of stem cell therapies without the associated transplantation risks. Most cell types secrete EVs that vary in size and cargo content and are capable of transferring signaling molecules like proteins, small RNAs, and DNA to target cells. Several studies have indicated that EVs from mesenchymal stem cells (MSCs) effectively mitigate cellular senescence in human and mouse cells (Fafian‐Labora et al. 2020; Kulkarni et al. 2018). In addition, our group demonstrated that EVs from young murine bone marrow‐derived MSCs or human MSCs differentiated from embryonic stem (ES) cells extended the lifespan and health span of mouse models of accelerated aging (Dorronsoro et al. 2021). Here we demonstrate that EVs derived from different types of functional stem cells such as embryonic progenitor cells, adipose‐derived MSCs, and human liver stem cells (Chiabotto et al. 2021) have senomorphic activity that results in significantly decreased senescence in IMR90 cells. Given that EVs are enriched with small RNAs including miRNAs, we examined whether miRNAs identified in EVs derived from multiple stem cells contribute to their senomorphic activity. Evaluation of miRNA profiles of five types of young stem cell EVs based on the number of their targeted aging‐related genes and prevalence in stem cells versus nonstem EVs identified eight miRNAs. Analysis of the miRNAs by transfection into etoposide‐induced senescent IMR90 cells revealed that each of the miRNAs alone regulated specific senescence and SASP markers, but none had complete senomorphic activity. We thus evaluated more than 300 combinations of miRNAs for senotherapeutic activity, identifying a cocktail of miR‐181a‐5p, miR‐92a‐3p, miR‐21‐5p, and miR‐186‐5p as having senomorphic activity, markedly reducing expression of *p16* ^*INK4a*^, *p21* ^*Cip1*^, *IL‐1β*, *and IL‐6* and the percentage of SA‐ß‐gal‐positive cells in cell culture and in vivo. RNA‐seq analysis demonstrated that these four miRNAs suppressed multiple signaling pathways including targeting PCAF and HIPK2 in the p53 signaling pathway. Overall, these studies suggest that a senomorphic miRNA cocktail identified in functional stem cell EVs could be used to target senescence and extend health span.

## 2 Materials and Methods

### 2.1 Cell Culture and Etoposide‐Induce Senescence

IMR90 cells were purchased from ATCC (Manassas, VA) and cultured in MEM—Minimum Essential Medium (Gibco) supplemented with 10% fetal bovine serum, 1% Sodium Pyruvate (Gibco), and 1% MEM nonessential amino acids (Gibco). All cells were maintained at 37°C in a humidified incubator with 5% CO_2_. Upon receiving the IMR90 cells from ATCC, they were cryopreserved in bulk to ensure uniformity across experiments by using cells of the same passage for each induction of cellular senescence. To induce senescence, IMR90 cells were treated with 20 μM Etoposide (Sigma) for 48 h. After 48 h of treatment, the etoposide was removed and replaced with fresh media. The medium was changed every 2 days for 6 days. Six days post etoposide removal, approximately 60% of IMR90 cells displayed senescent characteristics, as evidenced by positive SA‐β‐gal staining.

The miRNAs were transfected into etoposide‐induced senescent IMR90 cells to determine their senotherapeutic activity. mirVana miRNA mimics were purchased from Ambion. Cell transfections were performed using Lipofectamine RNAiMAX (Invitrogen) following the manufacturer's protocol.

### 2.2 Preparation and Quantification of Human Stem Cell‐Derived MSC Extracellular Vesicles

EVs were derived from various MSC sources, including mesenchymal‐like stem cells from human liver stem cells (HLSCs) characterized by their antifibrotic activity and delivery of specific miRNAs to target hepatic stellate cells (Chiabotto et al. 2021). Additionally, MSCs generated from human embryonic stem cells, including AC83, as well as two embryonic progenitor cell lines—RP1 (a pericyte progenitor) and E69 (a neural crest progenitor)—were employed to illustrate lineage‐specific EV content and function (Lee et al. 2023; Sternberg et al. 2014). These sources allow for the analysis of the functional diversity and regenerative capacity of EVs across multiple tissue contexts.

For isolation of EVs, HLSCs were seeded in hyperflasks (Corning, VWR International, Milano, Italy) at a density of 3000 cells/cm^2^, and MSCs were seeded in T150 flasks at 2000 cells/cm^2^. Both cell types were cultured under standard conditions until reaching 70%–80% confluence. EVs were isolated from the supernatants of HLSCs in serum‐free α‐MEM (Lonza) and MSCs in serum‐free RPMI‐1640 (Euroclone) after 18 h. The supernatant was collected, centrifuged at 3000 *g* for 15 min at 4°C, filtered through 0.22 μm filters to remove debris, and then transferred to polycarbonate bottles (Beckman Instruments). Sequential ultracentrifugation was performed at 10,000 *g* for 1 h and 100,000 *g* for 2 h at 4°C (Beckman Coulter Optima L‐100K). The EV pellet was resuspended in RPMI with 1% DMSO (Sigma‐Aldrich) and stored at −80°C until further use. All EV isolation processes follow standard ultracentrifugation techniques recommended by the International Society for Extracellular Vesicles (ISEV) and align with the Minimal Information for Studies of Extracellular Vesicles (MISEV) guidelines (Welsh et al. 2024).

EVs, diluted at least 1:200 in sterile saline, were analyzed using the NanoSight LM‐10 instrument (NanoSight Ltd., Amesbury, UK) with a 405 nm laser. For each EV sample, three 60‐s videos were recorded and analyzed using Nanoparticle Tracking Analysis Software (NTA v3.4) to determine EV concentration.

### 2.3 Characterization of Extracellular Vesicles

EV phenotype was assessed by flow cytometry using the MACSPlex Exosome Kit (Miltenyi Biotec, Germany) (Koliha et al. 2016). Approximately 2 × 10^9^ EVs were incubated with capture beads for 18 h at 450 rpm, followed by staining with APC‐conjugated anti‐CD63, anti‐CD81, and anti‐CD9 for 1 h. Samples were washed to remove unbound antibodies, and around 5000 beads per sample were analyzed on a Cytoflex flow cytometer (Beckman Coulter, USA) using CytExpert Software. Median fluorescence intensity (MFI) values were corrected by subtracting background MFI from a blank control.

Transmission electron microscopy (TEM) was performed to evaluate EV size and integrity. EVs of 3 × 10^9^ were placed on formvar carbon‐coated grids and left to adhere for 10 min before fixing with 2.5% glutaraldehyde and staining with NanoVan (Deregibus et al. 2016). Images were obtained using a Jeol JEM 1400 electron microscope (Jeol, Japan).

As previously published (Chiabotto et al. 2021), HLSC‐ and MSC‐derived EVs express exosomal markers CD9, CD63, CD81, and mesenchymal markers CD29, CD44, CD105 but lack HLA‐1, HLA‐DR, CD24, CD326, CD31, and CD45. The presence of exosomal markers TSG101 and CD63 was confirmed by Western blot and their nanosize vesicle integrity was confirmed by TEM.

### 2.4 Animal Models

Wild‐type male C57Bl/6 mice, 95 weeks old, were obtained from the National Institute on Aging (NIA) and housed under pathogen‐free and controlled temperature (22°C ± 2°C) and light conditions (12‐h light/12‐h dark cycle). All experimental procedures involving mice adhered strictly to the ethical guidelines and regulations stipulated by the Institutional Animal Care Committee (IACUC) at the University of Minnesota (Protocol # 2108‐39356A). To verify the senomorphic effect of AC83 EVs, the *Ercc1* ^−/Δ^ mouse model of accelerated aging and senescence was used. *Ercc1* ^−/∆^ mice were assigned randomly to either the control group (PBS) or AC83 group (*n* = 4/group) with two intraperitoneal (I.P.) Injections of 5 × 10^9^ AC83 EVs 1 week apart. Tissues were immediately collected for 4 days post second injection.

### 2.5 In Vivo E5 miRNA Mimics Treatment

To evaluate the effects of the E5 miRNA cocktail on senescence, all mice were divided randomly into two groups (*n* = 6/group): the control group and E5 miRNA cocktail (8 mg/kg) group. This cocktail was composed of miR‐181a‐5p, miR‐92a‐3p, miR‐21‐5p, and miR‐186‐5p, in a ratio determined by in vitro experiments to be 3:2:1:4, respectively. All mirVana miRNA mimics were purchased from Ambion with HPLC purification. The miRNA negative control and E5 miRNA mimics were prepared using an in vivo*‐jetPEI* transfection reagent (Polyplus Transfection, Illkirch, France) according to the manufacturer's instructions.

All mice were subjected to three intraperitoneal (I.P.) injections, with 2‐day intervals between each injection. Two days after the last injection, mice were euthanized and their tissues were immediately harvested and cryopreserved in liquid nitrogen for future gene expression and histological evaluation.

### 2.6 SA‐β‐Galactosidase Assay

To analyze SA‐β‐galactosidase (SA‐β‐Gal) activity through fluorescence, senescent and nonsenescent IMR90 cells were seeded in a black 96‐well plate at a density of 5000 cells/well, followed by transfection with miRNAs at 37°C and 5% CO_2_. The cells were then rinsed with PBS, followed by incubation with 100 nM Bafilomycin A1 for 1 h and subsequently with 100 μM fluorogenic substrate C_12_FDG for 2 h. After the C_12_FDG staining procedure, the cells were counterstained with 2 μg/mL Hoechst 33342 (Invitrogen) for 15 min. A high content fluorescent image acquisition and analysis platform Cytation 1 (BioTek) was used to quantitate the total number of viable cells (Hoechst staining) and the number of senescent cells (C_12_FDG positive cells). All images were analyzed in triplicate wells with nine fields per well to ensure comprehensive coverage of the well's surface.

### 2.7 Cell Viability and Caspase 3/7 Assay

Senescence or nonsenescent IMR90 cells were seeded in a 96‐well plate (5000 cells/well) and treated after 24 h with E5 cocktail. Cell viability and Caspase 3/7 activity were determined using the ATPase assay (Abcam) and Caspase‐Glo 3/7 Assay Systems (Promega), respectively.

### 2.8 SA‐β‐Gal Staining for Tissue

Frozen liver sections, from mice treated with the E5 miRNA cocktail and control, were cut at a thickness of 5 μm, followed by air‐drying on the slide and fixing in 10% formalin. Postfixation, the slides were incubated in SA‐β‐gal staining solution, which comprised 1 mg/mL 5‐bromo‐4‐chloro‐3‐indolyl‐beta‐d‐galactopyranoside (X‐gal), citric acid/sodium phosphate buffer, 5 mM potassium ferricyanide, 5 mM potassium ferrocyanide, 150 mM NaCl, and 2 mM MgCl_2_. The incubation took place at 37°C in a CO_2_‐free incubator for 16–18 h. After staining, the slides were washed with PBS and mounted with ProLong Gold Antifade Mountant with DAPI (Invitrogen) and air‐dried for 30 min.

For quantitative analysis (via DAPI staining), the slides were scanned at Cytation 1 image reader (BioTek). To get the full slide image of SA‐β‐gal staining, slides were scanned at the Panotiq Digital Slide Imaging System. To accurately distinguish the blue staining characteristic of SA‐β‐gal in tissue samples, as well as analyze the percentage of SA‐β‐gal‐positive cells, a pixel‐based quantification method was employed. This involved counting the total blue pixels (using Adobe Photoshop) and then normalizing this count to the total cell number.

### 2.9 Immunofluorescence Analysis of Tissue

The frozen liver slides were fixed with 4% formaldehyde in PBS for 10 min at room temperature. Following fixation, the slides were permeabilized with 0.1% Triton X‐100 and 1% bovine serum albumin (BSA) in PBS for 30 min at room temperature. After blocking with 5% BSA for 1 h, they were incubated with the primary antibody γ‐H2AX (1:200; Cell Signaling) at 4°C overnight. The next day, after thorough washing with PBS (three times), the cells were incubated with the secondary, Alexa Fluor 488‐conjugated anti‐rabbit antibody (1:200, Invitrogen) for 2 h at room temperature. Finally, the slides were scanned to identify and count the γ‐H2AX‐positive cells using the Cytation 1 Image Reader (BioTek).

### 2.10 Western Blotting

The cells were extracted in RIPA buffer (Cell Signaling Technology) with protease inhibitors (Roche), deacetylase Inhibitor (MedChemExpress), and phosphatase inhibitors (Cell Signaling Technology). The protein concentration was measured by Pierce BCA Protein Assay Kit and 25–50 μg of total lysate was loaded and immunoblotted for regular Western blot. The primary antibodies for PCAF (cat# 3378S), HIPK2 (cat# 5091S), Phospho‐p53 (Ser46; cat# 2521S), p53 acetyl K379 (cat# 2570S), and p53 (cat# 2527S) were purchased from Cell Signaling Technology.

### 2.11 RNA Extraction and Quantitative Real‐Time PCR

Total RNA and miRNA were extracted using the miRNeasy Mini Kit (Qiagen) according to the manufacturer's instructions. The concentration and purity of RNA were determined using a NanoDrop spectrophotometer (ThermoFisher). To evaluate mRNA expression, total RNA (0.5 μg) was converted to complementary DNA (cDNA) using the High‐Capacity cDNA Reverse Transcription Kit (Applied Biosystems) according to the manufacturer's instructions. For miRNA quantification, total RNA (50 ng) was converted to cDNA using a TaqMan MicroRNA Reverse Transcription Kit (Applied Biosystems) according to the manufacturer's protocol.

Quantitative real‐time PCR for mRNA and miRNA was performed using Powerup SYBR Green Master Kit (Applied Biosystems) or TaqMan Fast Advanced master mix (Applied Biosystems), respectively. miRNA expression was normalized to that of U6 snRNA, and GAPDH and 18S ribosomal RNA were used as the housekeeping genes to normalize qPCR data. All retro‐transcriptase (RT) reactions, including no‐template controls and RT minus controls, were run in a QuantStudio 5 PCR System (Applied Biosystems). Each assay was conducted in quadruplicate. Relative miRNA or mRNA expression was determined by the ^△△^Ct method.

### 2.12 IL‐6 ELISA Assay

Conditioned media from IMR90 cells treated with the E5 cocktail or control were collected and concentrated using Amicon Ultra‐15 centrifugal filters (Millipore). The concentrated samples were then diluted in sample diluent NS buffer, and the IL‐6 level was measured using a commercially available ELISA kit (Abcam) following the manufacturer's protocol.

### 2.13 RNA Library Preparation and Sequencing

Total RNA was isolated with the miRNeasy Mini Kit (Qiagen), and the quality of RNA was checked using a NanoDrop spectrophotometer (ThermoFisher). Samples were quantified by the University of Minnesota Genomics Center (UMGC) using fluorimetry (RiboGreen assay), and RNA was assessed using capillary electrophoresis (Agilent 4200 TapeStation). Total RNA samples were converted to Illumina sequencing libraries using Illumina's TruSeq RNA Sample Preparation Kit (Cat. # RS‐122‐2001) or Stranded mRNA Sample Preparation Kit (Cat. # RS‐122‐2101) and further sequenced on Novaseq S4 with 20 million reads per sample by UMGC.

### 2.14 RNA‐Sequencing and Bioinformatic Analysis

The preprocessing of raw‐sequencing reads entailed removing adapter sequences and low‐quality bases. Differential gene expression analysis was then conducted using edgeR within the CLC Genomics Workbench (CLCGWB) as integrated into the R/Bioconductor framework. Genes were classified as differentially expressed genes (DEGs) if they met the criteria of adjusted *p*‐values (false discovery rate; FDR) < 0.05 and an absolute fold change (FC) > 2.0. Pathway enrichment analysis was conducted using DAVID, based on the differentially expressed genes (DEGs). To further analyze these DEGs, Gene Set Enrichment Analysis (GSEA) was performed with default settings (1000 permutations for gene sets). The gene sets database employed was h.all.v2023.2.Hs.symbols.

DEGs were also analyzed by comparison with the SenMayo senescence gene set (125 genes). Heat maps were generated based on these selected genes to highlight the expression changes in senescence and apoptosis‐related pathways.

To delineate the unique gene expressions attributed to each microRNA, Venny^2.1^ was employed. The resulting data were then subjected to pathway enrichment analysis using DAVID, focusing on these unique expressions. The culmination of this analysis was visualized in the form of a heat map, illustrating the distinct enriched pathways associated with each microRNA.

### 2.15 Statistical Analysis

Results are expressed as mean ± standard error of mean (SEM) of three separate replicate experiments unless otherwise indicated. Statistical analysis was performed using GraphPad Prism 10. To assess the statistical significance of differences observed between the two groups, the Student's *t*‐test was employed. ANOVA was used to compare statistical differences among multiple groups. *p* values < 0.05 were considered statistically significant.

## 3 Results

### 3.1 Senomorphic Effects of Stem Cell‐Derived EVs on Cellular Senescence and Aging

**FIGURE 1:**
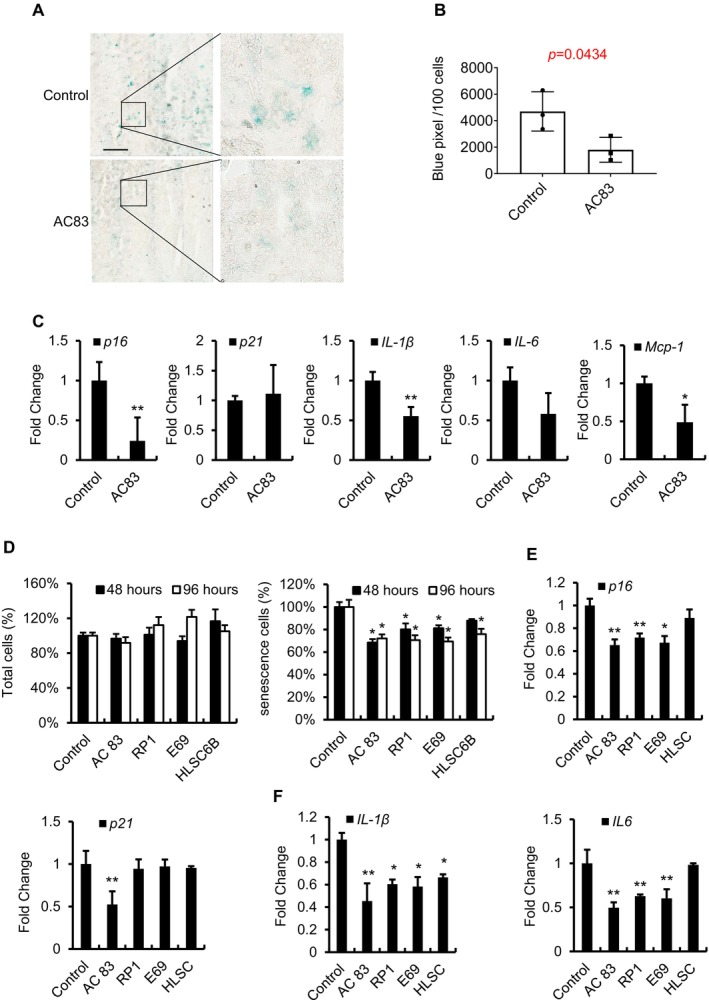
Evidence that multiple types of stem cell EVs function as senomorphics. (A) Representative image of SA‐β‐gal staining of liver sections from *Ercc1* ^*−*/Δ^ mice treated with AC83 EVs. Scale bar equals 2.5 mm. (B) Quantification of SA‐β‐gal staining of liver samples from *Ercc1* ^*−*/Δ^ mice treated with AC83 EVs. (C) qPCR analyses of *p16* ^*Ink4a*^, *p21* ^*Cip1*^, *IL‐1β*, *IL‐6*, and *Mcp‐1* of the livers from nature aging mice treated with AC83 EVs. (D) Quantification of total cell count and percentage of senescent IMR90 cells following treatment with four types of young stem cell‐derived EVs. Senescence was assessed by C12FDG SA‐β‐gal staining at 48 and 96 h posttreatment. Total cells were assessed using DAPI staining. Cell numbers are expressed as a percentage relative to untreated controls. (E, F) Relative mRNA expression of *p16* ^*Ink4a*^, *p21* ^*Cip1*^, *IL‐1β*, and *IL‐6* in senescent IMR90s as measured by qPCR. Data are shown as the mean ± SEM. P values are indicated with *p < 0.05 and **p < 0.01.

Our group previously demonstrated that EVs from murine bone marrow‐derived MSCs and MSCs derived from ES cells (AC83) had senomorphic activity in cell culture and in aged mice. We confirmed these results with AC83 EVs in both the *Ercc1* ^−/∆^ mouse model of accelerated aging and in naturally aged mice, observing a decrease in the percentage of SA‐β‐gal‐positive cells (Figures 1A,B and S1) and in the expression of *p16* ^*Ink4a*^ and SASP factors by RT‐PCR (Figure 1C), respectively. To determine if additional types of adult stem cell‐derived EVs have senomorphic activity, EVs from endothelial progenitor stem cells (RP1), embryonic progenitor stem cells (E69), and human liver stem cells (HLSCs) were examined for senotherapeutic activity on senescent IMR90s, with AC83 EVs used as a positive control. Interestingly, all these types of EVs were able to reduce the percentage of SA‐β‐gal‐positive cells, without significantly affecting the total cell counts, consistent with a senomorphic mechanism (Figure 1D). Quantitative PCR analysis further validated the senomorphic activity of the EVs, downregulating the expression of the key senescence‐related genes *p16* ^*INK4a*^ and SASP factors *IL‐6* and *IL‐1β* (Figure 1E,F). AC83 EVs also were able to downregulate *p21* ^*CIP1*^. These results collectively demonstrate the potent senomorphic capabilities of stem cell‐derived EVs.

### 3.2 Identification of Functional Senomorphic miRNAs in Stem Cell‐Derived EVs

**FIGURE 2:**
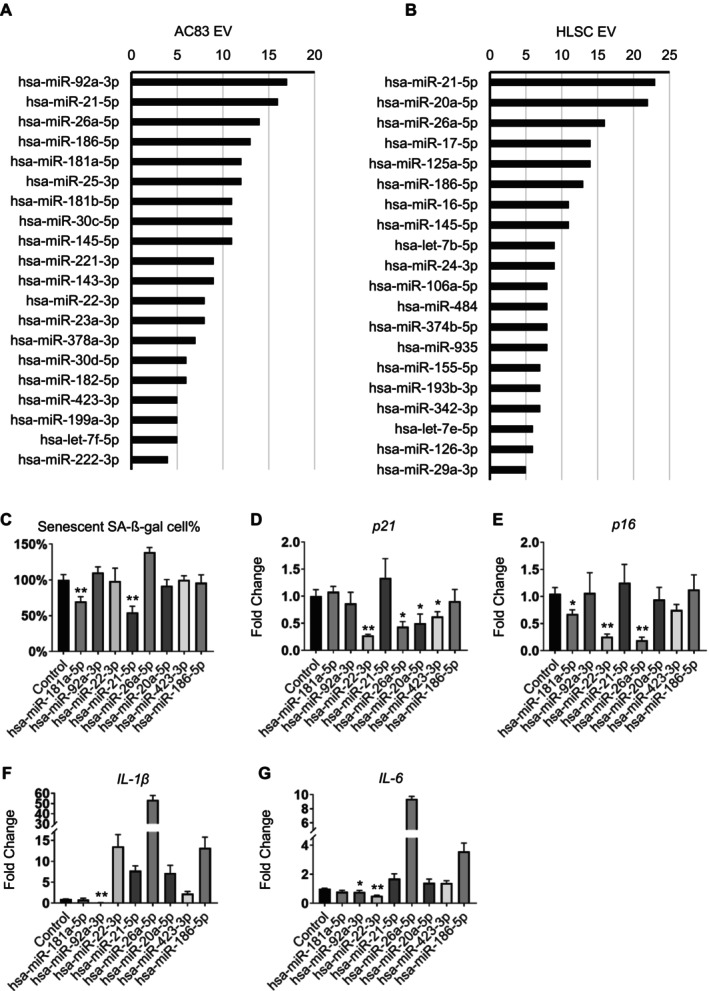
Identification of senomorphic miRNAs in young stem cell EVs. (A, B) Top 20 miRNAs from the miRNA profile of AC83 and HLSC. miRNAs were enriched using miEAA analysis and also scored based on the number of each miRNA targeted aging‐related genes from the GenAge database. (C) Percentage of SA‐β‐gal‐positive cells by C_12_FDG staining 96 h posttransfection of etoposide‐treated IMR90s with the indicated miRNAs. (D–G) Relative expression of *p21* ^*Cip1*^, *p16* ^*Ink4a*^, *IL‐1β*, and *IL‐6* as measured by qPCR. Data are shown as the mean ± SEM. P values are indicated with *p < 0.05 and **p < 0.01.

To identify potential senomorphic miRNAs in the stem cell‐derived EVs, we examined the five miRNA profiles of the functional stem cell EVs tested using the following criteria: (1) miRNAs present at high levels across five stem cell EV types and (2) miRNAs targeting the greatest number of aging‐related genes. Specifically, the miRNA Enrichment Analysis and Annotation Tool (miEAA) was used to enrich the top 40 miRNAs from the miRNA profiles. Then, the miRNAs were scored based on the number of targeted aging‐related genes as defined by the GenAge database. To further refine the selection, the background noise was minimized by subtracting the scores of miRNAs derived from normal fibroblast EVs (Figures 2A,B and S2). Using this screening approach, we identified eight candidate miRNAs that achieved high scores across all five of the functional stem cell EVs: miR‐181a‐5p, miR‐92a‐3p, miR‐22‐3p, miR‐21‐5p, miR‐26a‐5p, miR‐20a‐5p, miR‐423‐3p, and miR‐186‐5p.

To determine if these miRNAs had any senotherapeutic activity, they were transfected individually into etoposide‐induced senescent IMR90 cells and the percent of SA‐β‐gal‐positive cells as well as the expression of senescent and SASP markers were examined 96 h posttransfection. Although each miRNA affected some markers of senescence, no miRNA had full senomorphic activity. For example, miR‐181 reduced p16^INK4a^ expression and the proportion of SA‐β‐gal‐positive cells (Figure 2C,E), whereas miR‐21‐5p decreased the percentage of senescent cells but upregulated the expression of *IL‐1β* and *IL‐6* (Figure 2C,F,G). Similarly, miR‐26a‐5p and miR‐22‐3p significantly downregulated *p16* ^*INK4a*^ and *p21* ^*CIP1*^ but dramatically enhanced the level of *IL‐1β* (Figure 2D–F). In contrast, miR‐92a specifically reduced the levels of these SASP genes *IL‐1β* and *IL‐6* (Figure 2F,G). The varied effects on senescence and SASP markers observed from individual miRNAs led us to examine whether a combination of miRNAs would have a senomorphic activity like the parent EVs.

### 3.3 Optimization of a Senomorphic miRNA Cocktail

**FIGURE 3:**
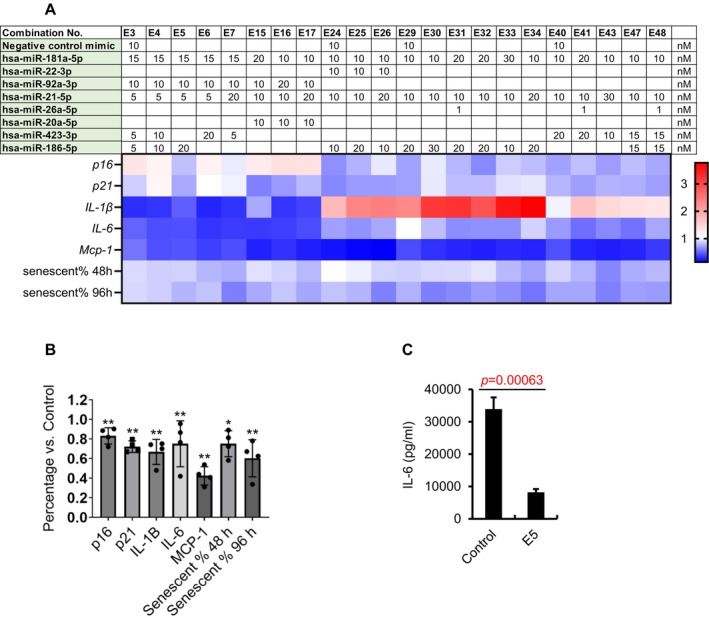
Identification of a senomorphic miRNA cocktail. (A) Screening of miRNA cocktails in senescent IMR90 cells. The table shows the concentration (nM) of each miRNA in different combinations (E3–E48). The heat map represents the expression levels of senescence markers (p16^Ink4a^, p21^Cip1^) and SASP factors (IL‐1β, IL‐6, Mcp‐1) by qPCR as well as the percentage of SA‐β‐gal‐positive cells at 48 and 96 h posttransfection. Blue indicates downregulation, and red indicates upregulation, with the scale showing fold‐change. (B) Expression of *p16* ^*Ink4a*^, *p21* ^*Cip1*^, and SASPs as well as the percent of SA‐β‐gal‐positive IMR90 cells after transfection of the E5 cocktail of miRNAs. (C) IL‐6 levels in the supernatant of senescent IMR90s 96 h posttransfection with the E5 cocktail. Data are shown as the mean ± SEM. *p* values are indicated with *p < 0.05 and **p < 0.01.

To identify an effective senomorphic miRNA, various doses and combinations of the eight miRNAs were tested in senescent IMR90 cells. The effectiveness of each combination was assessed by measuring the reduction in senescent cell numbers and the expression levels of key aging‐related genes, including *p16* ^*Ink4a*^, *p21* ^*Cip1*^, *IL‐1β*, *IL‐6*, and *Mcp‐1* 96 h posttransfection (Figures 3A and S3B–D). After multiple rounds of screening, we identified several combinations that reduced senescent cell counts and downregulated the expression of these genes. Further screening and optimization led to the identification of a combination of four miRNAs as the most effective senomorphic combination, termed E5 as the batch identifier, comprised of miR‐181a‐5p, miR‐92a‐3p, miR‐21‐5p, and miR‐186‐5p. Treatment of senescent IMR90s with the E5 cocktail resulted in a significant reduction in the expression of key aging markers, including *p16* ^*Ink4a*^, *p21* ^*Cip1*^, and SASPs, and decreased the percentage of SA‐β‐gal‐positive cells (Figure 3B) 96 h posttransfection. Additionally, E5 treatment did not affect caspase 3/7 activity or cell viability in either senescent or nonsenescent cells (Figure S3E,F) and had no impact on cell growth curves over time (Figure S3G,H), further confirming its senomorphic activity. Furthermore, the E5 cocktail effectively decreased IL‐6 levels in the cell culture supernatant (Figure 3C).

### 3.4 Elucidating the Senomorphic Mechanisms of E5 Cocktail

**FIGURE 4:**
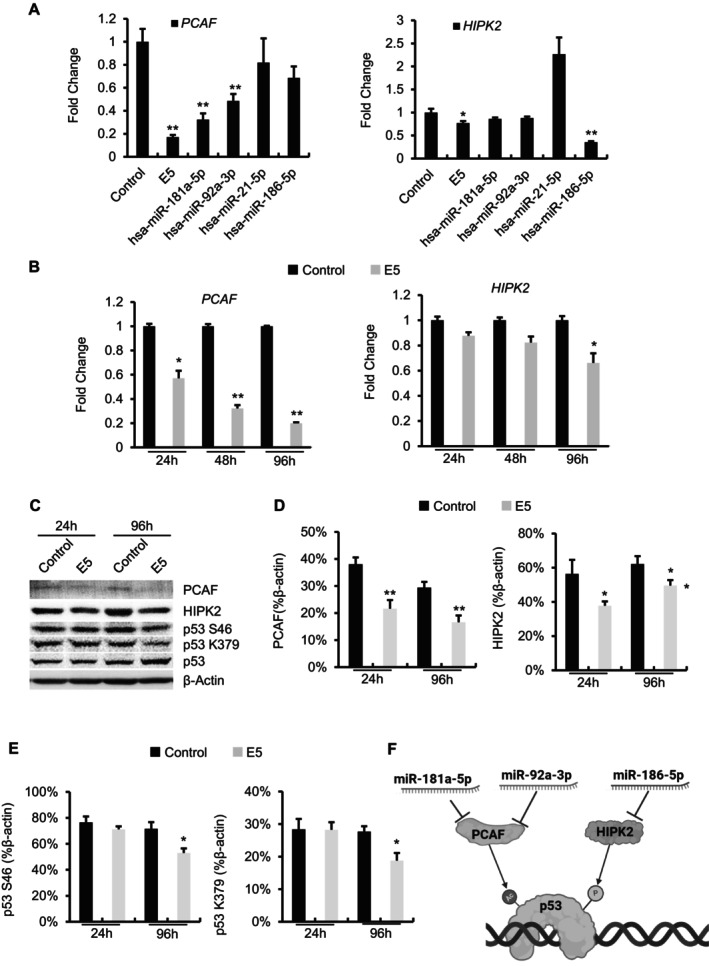
Possible senomorphic mechanism of E5. (A) Relative expressions of *PCAF* and *HIPK2* in senescent IMR90 cells quantitated by qPCR. (B) Expression of *PCAF* and *HIPK2* at 24, 48, and 96 h posttransfection with E5 by qPCR. (C) Representative immunoblots showing the protein levels of PCAF, HIPK2, p53 S46 (phosphorylated p53 at Ser46), and p53 K379 (acetylated p53 at Lys379) at 24 and 96 h posttransfection with E5. (D, E) Densitometry quantification of Western blot bands. (F) A model for how the E5 miRNA cocktail inhibits p53 activity. Data are shown as the mean ± SEM. P values are indicated with *p < 0.05 and **p < 0.01.

To examine the potential senomorphic mechanism of action of the E5 miRNA cocktail, we identified a set of 31 genes targeted by these miRNAs using miRwalk and the following selection criteria: (1) target genes of the E5 miRNAs in both the TargetScan and miRDB databases, (2) demonstrated upregulation during senescence in the SeneQuest.net database, and (3) evidence in the literature. This analysis revealed that E5 suppressed the expression of both *PCAF* and *HIPK2*, implicated in the regulation of senescence (Figure 4A). Furthermore, individual transfection of miR‐181a‐5p or miR‐92‐3p significantly decreased the expression levels of *PCAF*, whereas individual transfection of miR‐186‐5p markedly reduced the expression levels of *HIPK2* (Figure 4A). In addition, there was a consistent downregulation of *PCAF* at 24, 48, and 96 h posttransfection (Figure 4B). *HIPK2* plays a key role in facilitating p53 acetylation by *PCAF*, leading to the selective activation of *p21* ^*Waf1*^ in response to nonapoptotic DNA damage (Di Stefano et al. 2005; Kuwano et al. 2016). Immunoblot analyses corroborated the inhibitory effect of the E5 cocktail on the expression of *PCAF* and *HIPK2* (Figure 4C,D) and at different time points (Figure S4A,B). Additionally, we evaluated the effects of the E5 cocktail on p53 and its posttranslational modifications. Western blot analysis showed that E5 treatment significantly reduced phosphorylation of p53 at Ser46 (p53 S46) and acetylation at Lys379 (p53 K379) at 96 h posttransfection (Figures 4C,E and S4C). These findings are consistent with the role of HIPK2 and PCAF in mediating these specific modifications of p53. Taken together, these results demonstrate that the E5 cocktail not only affects the activity of *p53* but likely confers its senomorphic effects through multiple mechanisms.

### 3.5 Pathway Analysis of the E5 Cocktail

**FIGURE 5:**
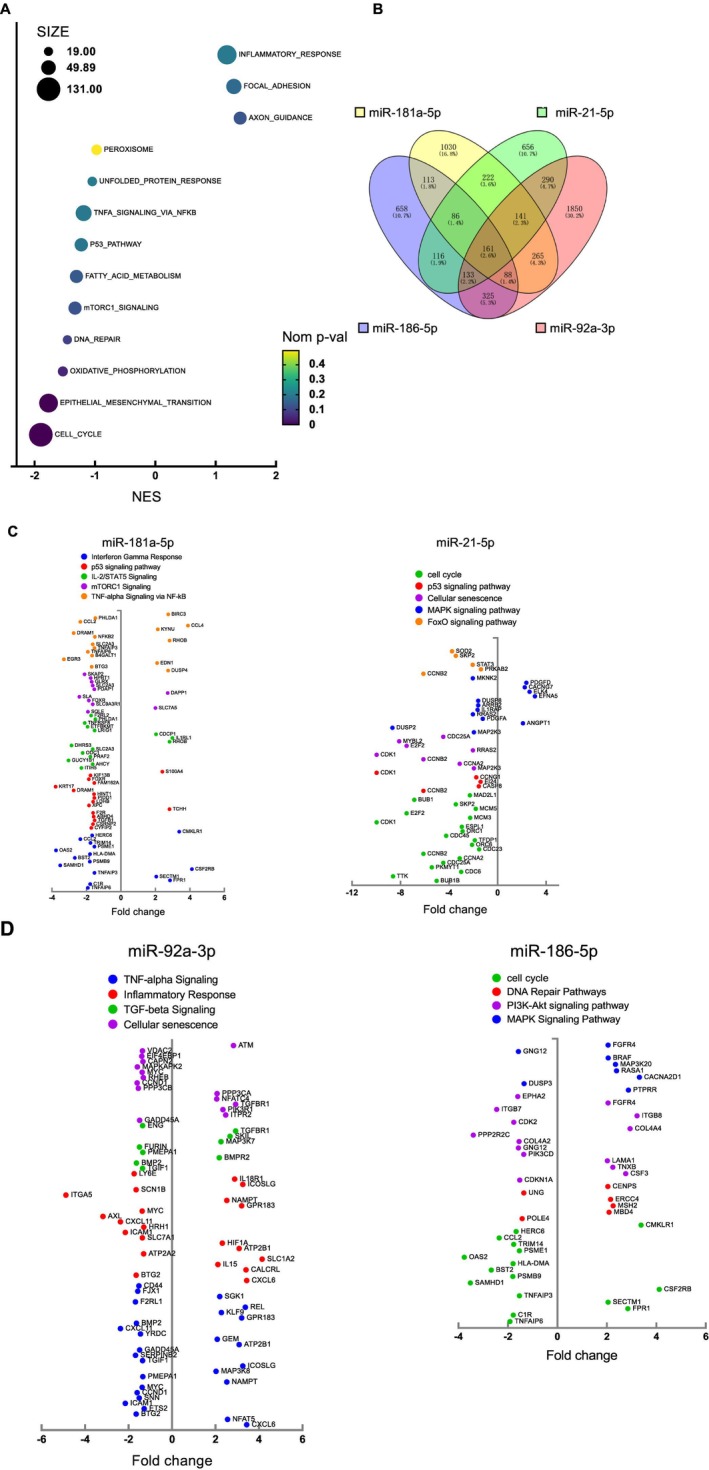
Pathways targeted by E5 cocktail. (A) Pathway enrichment analysis was conducted on RNA‐seq data using DAVID, based on the differentially expressed genes (DEGs) posttransfection with E5 cocktail. (B) Venn diagram of the unique gene expressions associated with each miRNA from the E5 cocktail. (C, D) Pathway enrichment analysis was conducted using DAVID, based on the unique gene expressions associated with each miRNA.

To further examine the senomorphic mechanisms of the E5 cocktail, we conducted RNA‐seq experiments on senescent IMR90s treated with the E5 cocktail or miR‐181a‐5p, miR‐92a‐3p, miR‐21‐5p, and miR‐186‐5p individually, each with three biological replicates. Pathway enrichment analysis using Gene Set Enrichment Analysis (GSEA) based on differentially expressed genes (DEGs) revealed that E5 markedly downregulated aging‐related pathways, including the cell cycle, mTORC1 signaling, and p53 signaling pathway (Figure 5A). To further investigate the mechanisms by which each E5 microRNA suppresses aging, we employed Venny (Oliveros 2015) to identify differentially expressed genes (DEGs) that are uniquely influenced by each microRNA compared to the other three microRNAs (Figure 5B). The pathway enrichment analysis for each miRNA is shown in Figure 5C,D, where individual miRNAs modulate specific pathways like p53, mTORC1 signaling, and TNF‐alpha signaling (miR‐181a‐5p), cell cycle and p53 signaling (miR‐21‐5p), and inflammation‐related (miR‐92a‐3p). These results further demonstrate that the E5 cocktail confers its senomorphic effect through multiple mechanisms.

### 3.6 Efficacy of E5 Cocktail in Natural Aging Models

**FIGURE 6:**
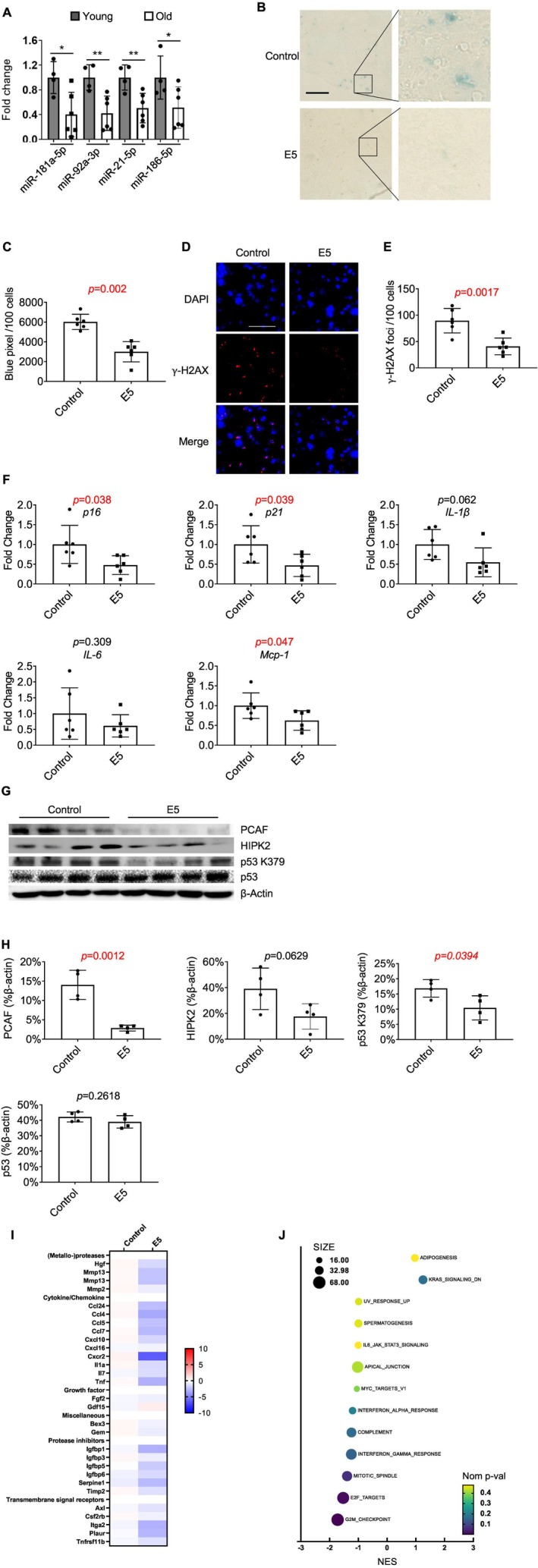
E5 Cocktail reduces senescence and SASP markers in naturally aged mice. (A) Endogenous expression of each miRNA in the liver of young and old mice. (B) Representative SA‐β‐gal staining images of liver section from aged mice, scale bar: 200 μm. (C) Quantification of SA‐β‐gal‐positive cells in the liver of old mice. (D) Representative immunofluorescence images showing γ‐H2AX‐positive cells in the liver of old mice (scale bar: 100 μm). (E) Quantification of γ‐H2AX‐positive cells in the liver of old mice. (F) Quantification of *p16* ^*Ink4a*^, *p21* ^*Cip1*^, IL‐1β, IL‐6, and *Mcp‐1* in the liver of aged mice. (G) Representative Western blots showing the expression levels of PCAF, HIPK2, p53, and acetylated p53 at Lys379 (p53 K379) in liver tissues of aged mice treated with the E5 cocktail or control. (H) Densitometry quantification of the Western blot bands for PCAF, HIPK2, p53, and p53 K379, normalized to β‐Actin (*n* = 4 per group). (I) Heatmap displaying differentially expressed genes from the SenMayo senescence gene set in the liver of aged mice treated with the E5 cocktail. (J) Gene Set Enrichment Analysis (GSEA) of liver tissue from aged mice treated with the E5 cocktail. Pathways were identified as significantly enriched based on RNA‐seq data and are displayed according to their normalized enrichment score (NES) and nominal *p*‐value (Nom p‐val). Data are shown as the mean ± SEM. P values are indicated with *p < 0.05 and **p < 0.01.

To examine whether the levels of miR‐181a‐5p, miR‐92a‐3p, miR‐21‐5p, and miR‐186‐5p are altered with age, their expression in the liver tissue of aged mice was compared to young mice. Interestingly, the expression of all four miRNAs declined with age in liver tissue (Figure 6A). To determine if the E5 cocktail had senomorphic activity in vivo, similar to stem cell‐derived EVs, the effect of liposome‐mediated delivery of the E5 miRNAs on the senescent cell burden was examined in 2‐year‐old C57BL/6 mice. The mice were treated with three intraperitoneal (I.P.) injections over a 2‐week period, and tissues were harvested for analysis. We validated the transfection efficiency of the four mimics through qPCR analysis of the liver in the treated mice (Figure S6D). Treatment with the E5 cocktail resulted in a reduction in the percentage of SA‐β‐gal‐positive cells (Figure 6B,C) and γ‐H2AX‐positive cells (Figure 6D,E) in the liver. Similarly, there was a consistent reduction in the expression of senescence‐associated genes, such as *p16* ^*Ink4a*^, *p21* ^*Cip1*^, and *Mcp‐1*, and trends toward lower *IL‐1β* and *IL‐6* levels (Figure 6F). At the protein level, western blot analysis of liver tissues showed that E5 treatment significantly reduced PCAF expression and moderately decreased HIPK2 levels, while also reducing acetylation of p53 at Lys379 (p53 K379), a modification linked to its transcriptional activity (Figure 6G,H). These results align with the in vitro findings, suggesting that E5 modulates p53 activity via suppression of PCAF and HIPK2. Additionally, several senescence markers show a significant decrease in the kidney and spleen, further validating the senomorphic potential of the E5 miRNA cocktail (Figure S6B,C). To further investigate the underlying mechanisms of E5 activity in vivo, bulk RNA‐seq analysis of liver tissue was performed. Treatment with the E5 cocktail reduced the expression of the SenMayo senescence gene set, particularly in genes linked to cytokine signaling, metalloproteases, and growth factor pathways (Figure 6I). In addition, the RNA‐seq analysis revealed that E5 treatment primarily influenced pathways involved in inflammation suppression and cell cycle regulation (Figure 6J), consistent with the senomorphic activity of the E5 miRNAs. Together, these findings suggest that liposome‐or vesicle‐mediated delivery of the E5 miRNAs is an effective strategy to reduce the senescent cell burden.

## 4 Discussion

We previously demonstrated that EVs from murine and human MSC sources exhibited senomorphic effects on senescent cells. Here we demonstrate that additional types of stem cells, including two types of embryonic progenitors and human liver stem cells, produce EVs able to function as senomorphics. We identified eight miRNAs, miR‐181a‐5p, miR‐92a‐3p, miR‐22‐3p, miR‐21‐5p, miR‐26a‐5p, miR‐20a‐5p, miR‐423‐3p, and miR‐186‐5p, within these stem cell‐derived EVs that suppress markers of senescence and SASP. Many of these miRNAs have already been demonstrated to be significantly downregulated during aging, such as miR‐181a‐5p (Saccon et al. 2021; Guo et al. 2016; Lu et al. 2021), miR‐21‐5p (Rippe et al. 2012), miR‐92a‐3p (Rippe et al. 2012; Ohyashiki et al. 2011), miR‐22‐3p (Sequeira and Godad 2023), miR‐26a‐5p (Sequeira and Godad 2023), miR‐186‐5p (Kim et al. 2016), and miR‐20a‐5p (Wang et al. 2011; Hackl et al. 2010) and we confirmed that the expression of miR‐181a‐5p, miR‐92a‐3p, miR‐21‐5p, and miR‐186‐5p indeed was reduced in the liver of aged mice. It is important to note that there may be additional miRNAs that contribute to the EV senomorphic activity that were not tested.

Following transfection into senescent IMR90 cells, each miRNA displayed unique partial senomorphic effects, with some reducing senescence but simultaneously increasing SASP gene expression. For example, miR‐181a‐5p reduced aging markers, miR‐21‐5p decreased senescence but increased inflammatory genes, whereas others like miR‐26a‐5p both reduced and increased specific aging markers. The fact that senescence and SASP markers were affected differently by the miRNAs is of interest since it suggests that p16^INK4A^, p21^CIP1^, and SA‐ß‐gal markers of senescence can be separated. miR‐21‐5p decreased the percent of SA‐ß‐gal‐positive cells but marginally increased the expression of *p16* ^*INK4A*^ and *p21* ^*CIP1*^ and significantly induced *IL‐1ß* and *IL‐6*. In contrast, miR‐26a‐5p was able to reduce p16^INK4A^ and p21^CIP1^ expression while increasing the percent of SA‐ß‐gal‐positive cells and dramatically increasing the expression of IL‐1ß and IL‐6. Of all the miRNAs tested, only miR‐92a‐3p was able to suppress *IL‐1ß* expression while having no effect on senescence markers.

The analysis of the effects of each individual miRNA on senescence elucidated the necessity of combining multiple miRNAs into a cocktail to enhance senomorphic activity. After several rounds of screening of different miRNA combinations and doses, we selected a combination of miR‐181a‐5p, miR‐92a‐3p, miR‐21‐5p, and miR‐186‐5p (E5) for future studies. Although the miRNA profile in the EVs suggests a ratio of miR‐181a‐5p, miR‐92a‐3p, miR‐21‐5p, and miR‐186‐5p that is approximately 5:3:1:1, in our in vivo experiments, we used a ratio of 3:2:1:4 based on the transfection results. Thus, the ratios of miRNAs used for liposome delivery were comparable to the EVs except for the increased dose of miR‐186‐5p.

The effects of miR‐92a‐3p inhibition on hypoxia/reoxygenation was shown previously to be mediated by *Smad7* (Zhang et al. 2014). miR‐181a‐5p was implicated in cellular senescence via its downregulation of *SIRT1* (di Rivetti Val Cervo et al. 2012), a member of the sirtuin family able to modulate senescence (Chen et al. 2020). Additionally, miR‐186‐5p targets BACE1, the crucial enzyme in β‐amyloid formation, suggesting that this miRNA plays a key role in Alzheimer's disease (Kim et al. 2016; Ben Halima et al. 2016). Among the four miRNAs studied, miR‐181a‐5p and miR‐21‐5p demonstrated efficacy in reducing senescent cell number, whereas miR‐92a‐3p was effective in reducing the expression of SASP genes. Interestingly, despite not impacting traditional aging markers and even increasing SASP gene expression, miR‐186‐5p proved to be an essential component of the E5 cocktail.

To explore the senomorphic mechanisms of the E5 cocktail, we chose 31 target genes of E5 miRNAs based on several selection criteria. This analysis identified a possible mechanism of the collaborative action of miR‐186‐5p, miR‐92a‐3p, and miR‐181a‐5p on inhibiting the activity of p53 via the downregulation of *PCAF* and *HIPK2*. This hypothesis is supported by published literature, suggesting miR‐186‐5p suppresses HIPK2 in cooperation with METTL3 and miR‐221‐3p (Cui et al. 2020; Pan et al. 2021). Additionally, PCAF has been identified as a target gene of both miR‐92a‐3p (Zhao et al. 2012) and miR‐181a‐5p (Zhao et al. 2012; Pichiorri et al. 2008). Our analysis (Figure S4A,B) also suggested that E5 miRNAs directly target the expression of *DRAM* (damage regulated autophagy modulator 1) involved in p53‐induced autophagy (Crighton et al. 2006), *IL‐18*, a key player in inflammation (Dinarello 2006) and fibroblast senescence (Zhang et al. 2019), and *MOAP1* (modulator of apoptosis 1), a Bax‐associated protein‐mediating caspase‐dependent apoptosis. In addition, RNA‐seq analysis demonstrated that each E5 miRNA affected aging‐related pathways in unique ways. For example, miR‐21‐5p predominantly downregulates the cell cycle and MAPK signaling pathways, whereas miR‐92a‐3p primarily targets inflammation‐related pathways. Also, both miR‐21‐5p and miR‐92a‐3p modulate the DAVID‐defined cellular senescence pathway.

The studies in naturally aged mice confirm the E5 cocktail's senomorphic effect in the liver, kidney, and spleen of aged mice. In particular, bulk RNA‐seq analysis of the liver demonstrated a reduction in the SenMayo panel of genes as well as in inflammatory and cell cycle regulatory pathways. Additionally, the reduced expression levels of each of the E5 miRNAs in the liver of aged mice highlight the importance of supplementing these senomorphic miRNAs. It is important to note that there are differences between EVs and liposome delivery systems. While EVs offer natural targeting capabilities, liposomes require optimization for comparable efficacy and cell type targeting. However, the fact that the E5 cocktail works effectively in vivo clearly documents the senomorphic activity of these miRNAs.

As this manuscript was being revised, a publication reported the ability of human embryonic stem cell‐derived exosomes (hESC‐Exos) to reverse senescence and restore the proliferative capacity of SnC in cell culture as well as extended health span and lifespan in mice. At least part of the hESC‐Exos effect appears to be mediated by miR‐302b, enriched in hESC‐Exos, that targets the cell cycle inhibitors Cdkn1a and Ccng2 (Bi et al. 2025). However, miR‐302b is not present in the progenitor stem cell‐derived EVs used in our study. In addition, our preliminary studies suggest that iPSC‐derived EVs may be more rejuvenating in aged cells than senotherapeutic compared to MSC‐derived EVs. We also have identified a senolytic miRNA, miR‐106b‐5p, that is expressed at a higher level in centenarians and in Ames Dwarf mice. Clearly, there are multiple miRNAs able to modulate senescence and extend health span.

In conclusion, these results document that many different types of stem cells release senomorphic EVs capable of suppressing markers of senescence and SASPs. All the senomorphic EVs contained at least four miRNAs, miR‐181a‐5p, miR‐92a‐3p, miR‐21‐5p, and miR‐186‐5p, which we demonstrated are important for a senomorphic effect in cell culture and in vivo. These miRNAs target key aging‐related pathways as well as specific genes, such as *PCAF*, *HIPK2*, *DRAM*, *IL‐18*, and *MOAP1*. This study not only emphasizes the potential of miRNA‐based therapies to reduce senescence with its associated inflammatory SASP but also identifies an effective senomorphic combination of miRNAs that could be used clinically to reduce senescence and inflammation to extend health span.

## Author Contributions

T.Z. and A.D.C.N. performed the majority of the experiments and wrote the first draft; J.L., D.L., G.C., and S.K.L. provided extracellular vesicles; V.U.B., L.A., R.D.O., and L.J.N. helped with the mouse experiments; X.D. assisted with the bioinformatics; and P.D.R. designed the experiments and edited the manuscript.

## Conflicts of Interest

J.L. and D.L. are employees of AgeX, G.C. is a co‐founder of Unicyte GmbH, and S.K.L. is a co‐founder of Paracrine Therapeutics. L.J.N. and P.D.R. are co‐founders of Itasca Therapeutics and inventors on several senescence‐related patents owned by the University of Minnesota.

## Supporting information


**Figure S1.** Supplementary images of SA‐β‐gal staining of liver sections from *Ercc1* ^*−*/Δ^ mice treated with AC83 EVs; scale bar is set to 2.5 mm.
**Figure S2.** Comparative quantification of miRNA profiles derived from fibroblast EV, E69 EV, RP‐1 EV, and MSC EV.
**Figure S3.** (A) qPCR assessment of miR‐181a‐5p, miR‐92a‐3p, miR‐186‐5p, and miR‐21‐5p expression levels at 96 h following transfection with 50 nM of each corresponding miRNA mimic in senescent IMR90 cells. (B–D) Additional evaluations of different combinations of miRNAs in senescent IMR90 cells. (E) Caspase 3/7 activity in nonsenescent and senescent IMR90 cells treated with E5 at 24, 48, and 96 h. (F) Relative viability of nonsenescent and senescent IMR90 cells treated with E5 at 24, 48, and 96 h. (G) Growth curves of non‐senescent cells treated with the E5 cocktail. Cell growth was monitored over 6 days posttreatment, and results are expressed as the fold change relative to Day 1. (H) Growth curves of senescent cells treated with the E5 cocktail. Cell growth was monitored over 16 days of posttreatment, and results are expressed as the fold change relative to Day 1. Data are shown as the mean ± SEM of three independent experiments.
**Figure S4.** (A) Quantitative analysis of the relative expression levels of *DRAM*, *IL‐18*, and *MOAP1* as determined by qPCR. (B) Time‐course qPCR analysis detailing the expression patterns of *DRAM*, *IL‐18*, and *MOAP1* at 24, 48, and 96 h after transfection with the E5 miRNA cocktail. (C) The densitometer quantification of p53 at 24 and 96 h posttransfection with E5. Data are shown as the mean ± SEM. **p < 0.01.
**Figure S5.** (A) Enrichment analysis of pathways regulated by each miRNA using DAVID based on their target genes. None‐age and disease related pathways were removed. The size of the diamonds indicates the target genes number. Yellow diamonds represent inflammation‐related pathways, blue for stress response‐related pathways, and green signifies pathways related to cell proliferation, survival, growth, migration, and differentiation.
**Figure S6.** (A) Additional images of SA‐β‐gal staining of liver sections from nature aging mice treated with the E5 cocktail. The scale bar is set to 200 μm. (B, C) qPCR analyses of *p16* ^*Ink4*^, *p21* ^*Cip1*^, *IL‐1β*, *IL‐6*, and *MCP‐1* in the kidney (B) and spleen (C) of naturally aged mice. (D) Expression of E5 miRNA mimics in the liver of naturally aged mice, after three intraperitoneal (I.P.) injections administered at 2‐day intervals. Data are shown as the mean ± SEM.

## Data Availability

All RNA‐seq data are available upon request.
