# Supplementary material for: Identification of Senomorphic miRNAs in Embryonic Progenitor and Adult Stem Cell‐Derived Extracellular Vesicles
Source: Aging Cell. 2025 Apr 24;24(7):e70071. doi: 10.1111/acel.70071 (PMC12266766; doi:10.1111/acel.70071)

**Supplemental Figure 1**

4X

Control

2.5mm

AC83


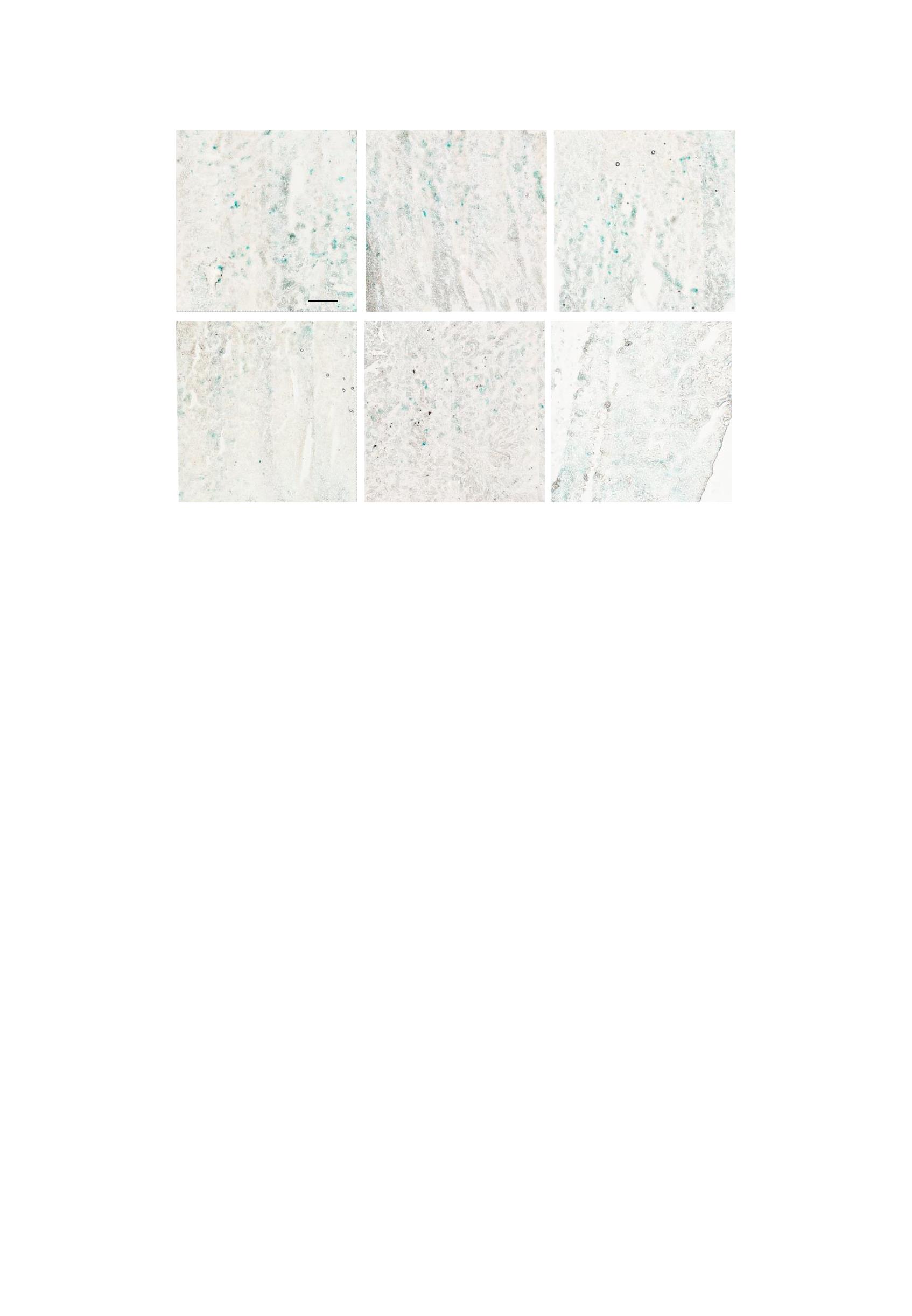


**Supplemental Figure 2**

Fibroblast EV

E69 EV

20

0

10

30

40

0

5

10

15

20

25

hsa-miR-93-5p

hsa-miR-20a-5p

hsa-miR-20b-5p

hsa-miR-21-5p

hsa-miR-17-5p

hsa-miR-15a-5p

hsa-let-7a-5p

hsa-miR-16-5p

hsa-miR-155-5p

hsa-miR-17-5p

hsa-miR-193b-3p

hsa-miR-106a-5p

hsa-miR-484

hsa-miR-320a-3p

hsa-let-7b-5p

hsa-miR-130b-3p

hsa-let-7c-5p

hsa-miR-24-3p

hsa-miR-146a-5p

hsa-miR-100-5p

hsa-miR-222-3p

hsa-miR-19b-3p

hsa-miR-149-5p

hsa-miR-29a-3p

hsa-miR-331-3p

hsa-miR-197-3p

hsa-miR-454-3p

hsa-miR-1225-3p

hsa-miR-214-3p

hsa-miR-22-3p

hsa-miR-27a-3p

hsa-miR-26a-5p

hsa-let-7g-5p

hsa-miR-130a-3p

hsa-miR-145-5p

hsa-miR-106a-5p

hsa-miR-143-3p

hsa-let-7b-5p

hsa-miR-27b-3p

hsa-let-7i-5p

RP-1 EV

MSC EV

0

10

20

30

40

0

5

10

15

20

25

hsa-miR-93-5p

hsa-miR-20a-5p

hsa-miR-20b-5p

hsa-miR-21-5p

hsa-miR-17-5p

hsa-miR-29b-3p

hsa-let-7a-5p

hsa-miR-125b-5p

hsa-miR-21-5p

hsa-miR-30a-5p

hsa-miR-320a-3p

hsa-miR-320a-3p

hsa-miR-125a-5p

hsa-miR-30c-5p

hsa-miR-26a-5p

hsa-miR-145-5p

hsa-miR-186-5p

hsa-miR-16-5p

hsa-let-7b-5p

hsa-let-7c-5p

hsa-miR-320a-3p

hsa-miR-22-3p

hsa-miR-26a-5p

hsa-miR-146a-5p

hsa-miR-27a-3p

hsa-miR-106a-5p

hsa-let-7b-5p

hsa-miR-24-3p

hsa-miR-138-5p

hsa-miR-30b-5p

hsa-miR-193b-3p

hsa-miR-484

hsa-miR-29c-3p

hsa-miR-222-3p

hsa-miR-130a-3p

hsa-miR-100-5p

hsa-miR-17-5p

hsa-miR-374b-5p

hsa-miR-221-3p


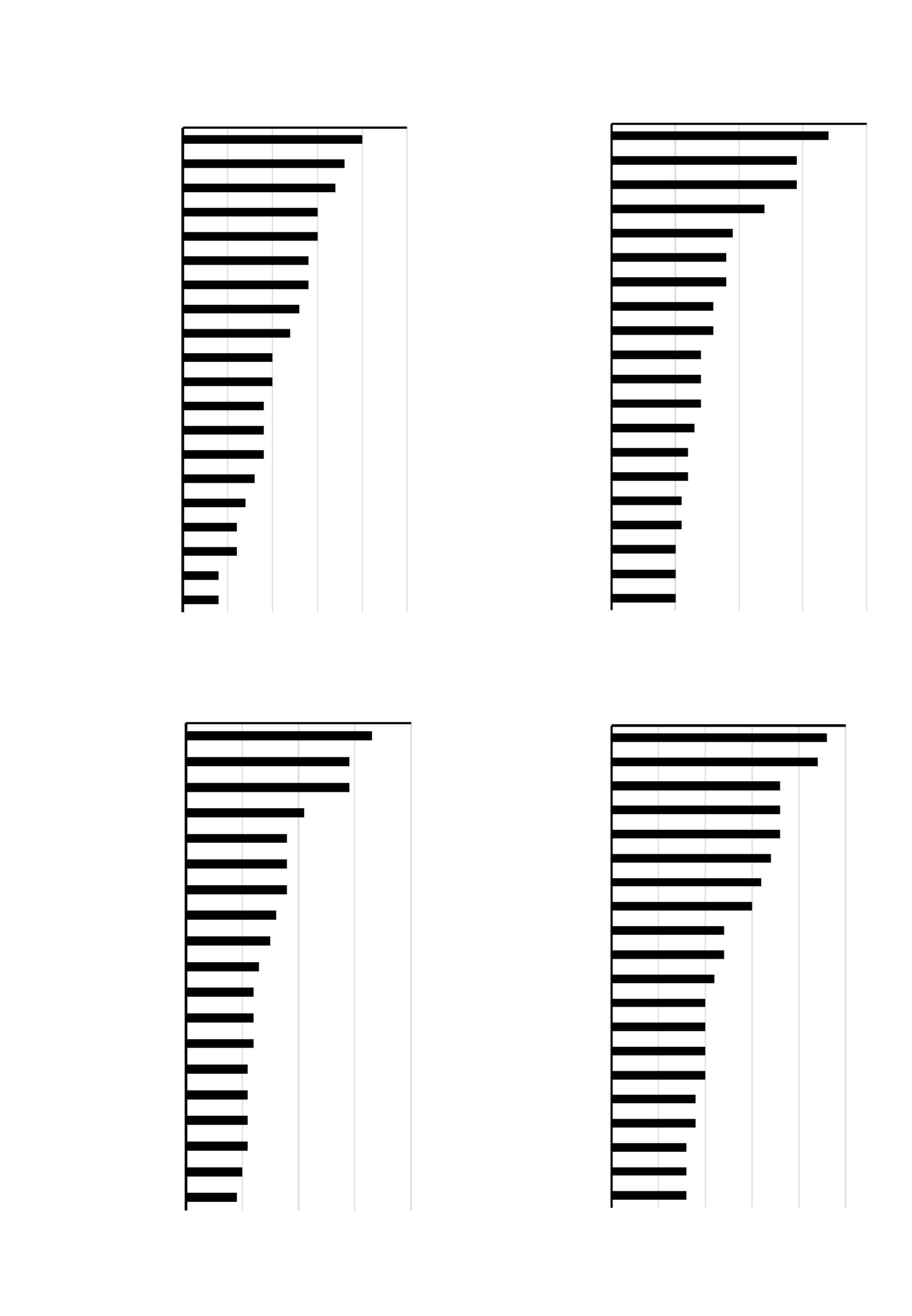


**Supplemental Figure 3**

**A**

miR-181a-5p

miR-92a-3p

miR-186-5p

3500

3000

2500

2000

1500

1000

500

100000

90000

80000

70000

60000

50000

40000

30000

20000

10000

0

4000

3500

3000

2500

2000

1500

1000

500

**

**

**

0

0

miR-21-5p

1200

1000

800

600

400

200

0

**

**B**

**Combination No.**

**Negative control mimic**

**hsa-miR-181a-5p**

**hsa-miR-22-3p**

**hsa-miR-92a-3p**

**hsa-miR-21-5p**

**hsa-miR-26a-5p**

**hsa-miR-20a-5p**

**hsa-miR-423-3p**

**hsa-miR-186-5p**

**C2 C3 C4 C5 C6 C7 C8 C9 C10 C11 C12 C14 C16 C17 C27 C28 C29 C30 C31 C32**

10

nM

10 10 10 10 nM

12.0 10 10 10 10 10 10 10 10 10 10 10 10 10

10.0

7.5

10

10 10 10 10 20 10 10 20

10 10

10

10

nM

nM

10

20

2.5 10 10 10 10 10 10 10 10 10 10 10

2.5 10 10

1.0 10 10 10 10 10 10

10 10

10

10 10 10 10

10

10 nM

nM

nM

2.5

2.5

10

10

20 20 nM

nM

10

10

20 20

*p16*

*p21*

3

2

1

*IL-1β*

*IL-6*

*Mcp-1*

senescent% 96h


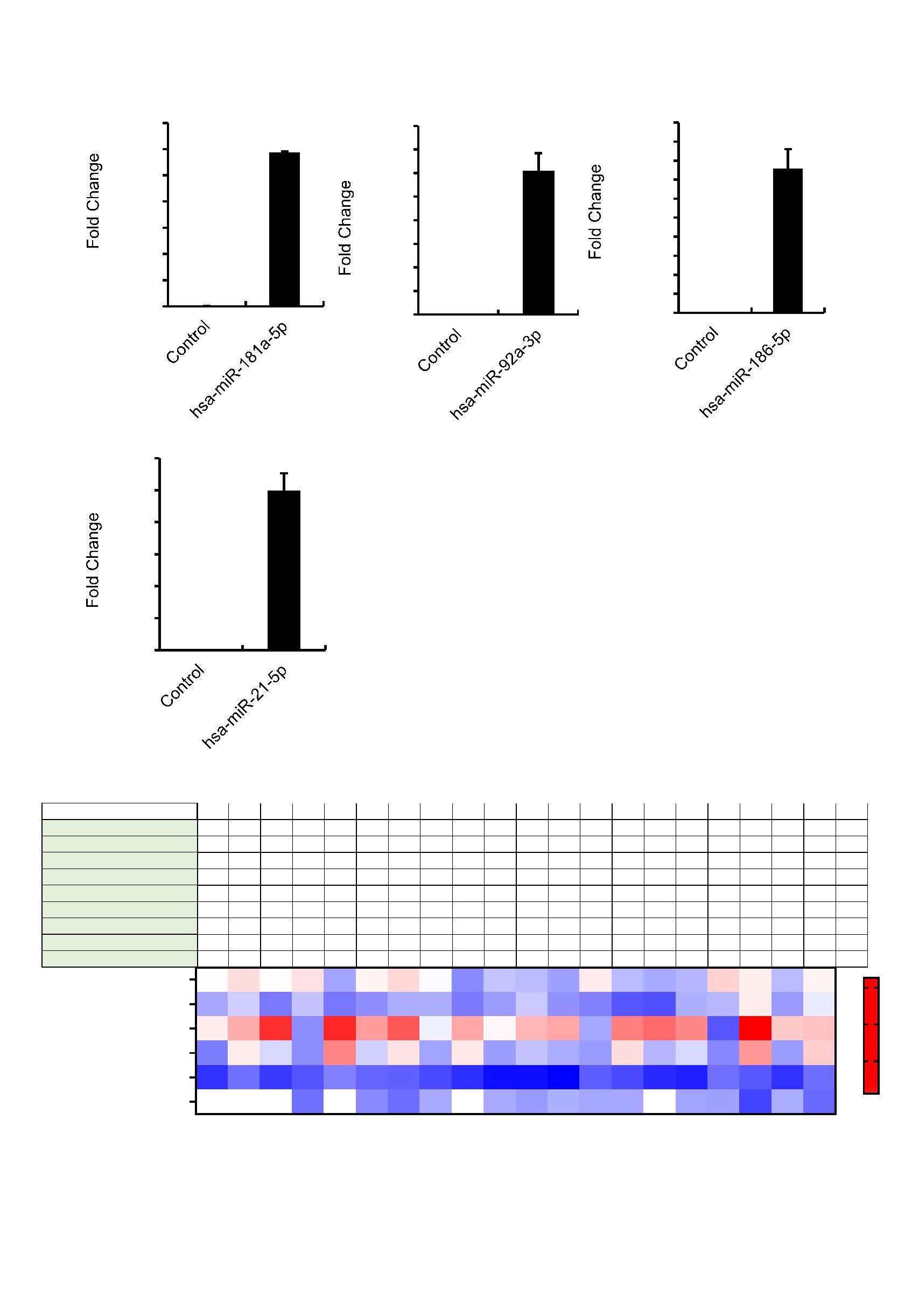


**Supplemental Figure 3**

**C**

**Combination No.**

**G2 G5 G7 G9 G12 G18 G22 G23 G33**

**Negative control mimic**

**hsa-miR-181a-5p**

**hsa-miR-22-3p**

**hsa-miR-92a-3p**

**hsa-miR-21-5p**

**hsa-miR-26a-5p**

**hsa-miR-20a-5p**

**hsa-miR-423-3p**

**hsa-miR-186-5p**

nM

15 15 15 25 20 20 10 10 20 nM

10

nM

10 nM

10 10 10 10

5

5

1

5

1

5

10 10 30 30 20 nM

1

nM

nM

nM

nM

20 10

5

5

20 20 10 10 20

*p16*

*p21*

9

7

5

3

1

*IL-1β*

*IL-6*

*Mcp-1*

senescent% 48h

senescent% 96h

**D**

**Combination No.**

**J2 J3 J10 J14 J18 J19 J20 J21 J25 J32**

**Negative control mimic**

nM

20 20 20 15 25 20 25 30 10 10 nM

**hsa-miR-181a-5p**

**hsa-miR-22-3p**

**hsa-miR-92a-3p**

**hsa-miR-21-5p**

**hsa-miR-26a-5p**

**hsa-miR-20a-5p**

**hsa-miR-423-3p**

**hsa-miR-186-5p**

10

nM

10 nM

10

5

10 10 10 10

10 10 10

1

5

5

5

1

5

1

10 25 nM

1

nM

nM

nM

nM

15

15

5

20 10

20 10 15 10 10 15

*p16*

*p21*

16

11

6

*IL-1β*

*IL-6*

*Mcp-1*

senescent% 48h

senescent% 96h

1

**E**

**F**

300000

140%

24H

48H

96H

24H

48H

96H

120%

100%

80%

60%

40%

20%

0%

250000

200000

150000

100000

50000

0

Control

E5

Control

E5

Control

E5

Control

E5

Non-senescent

Senescent

Non-senescent

Senescent


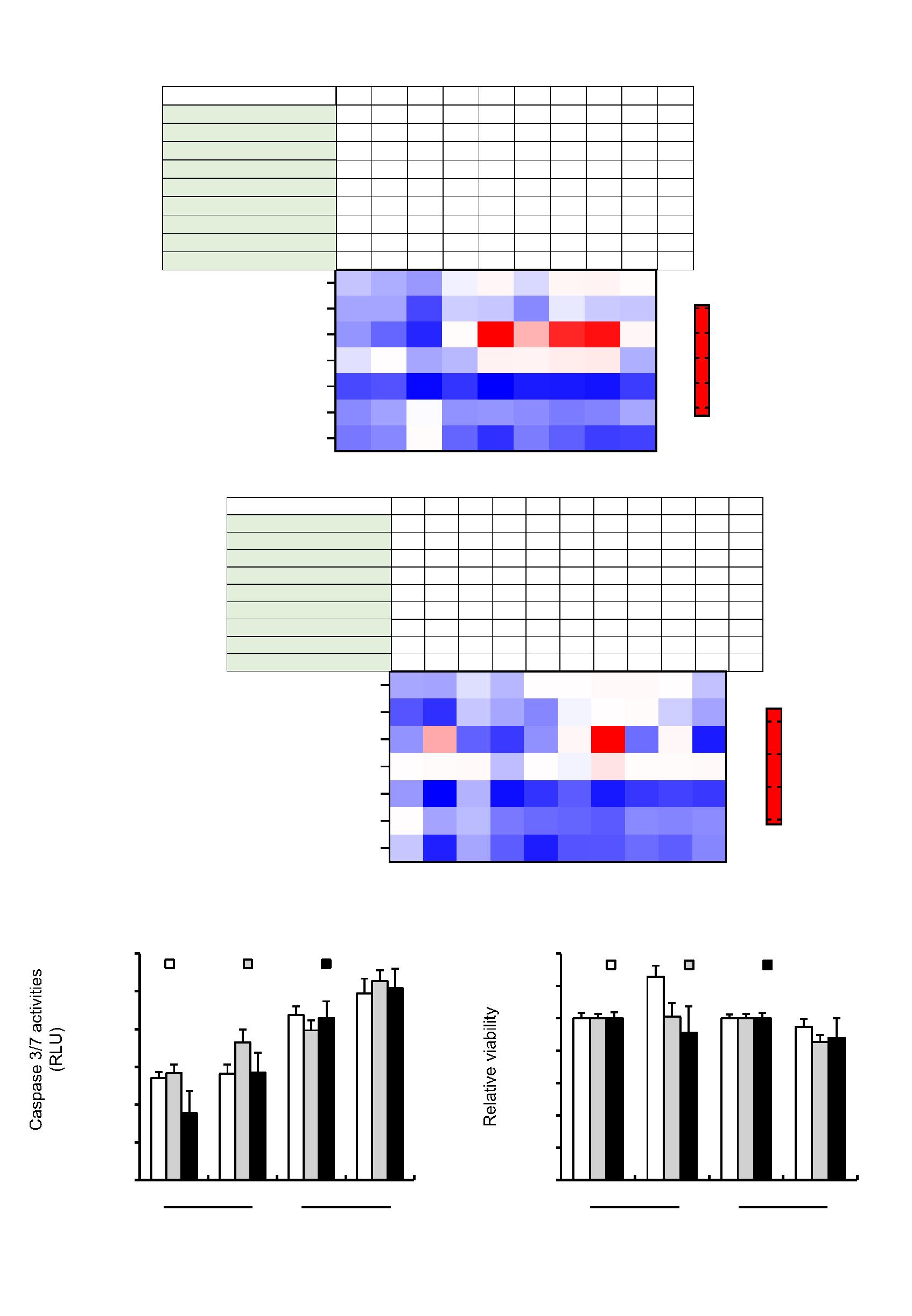


**G**

**H**

1.2

1.0

0.8

0.6

control

E5

5

4

3

2

1

0

control

E5

Day 1 Day 2 Day 4 Day 6

1

2

4

0

6

1

1

y

y

y

a

a

a

y

y

D

D

D

a

a

D

D


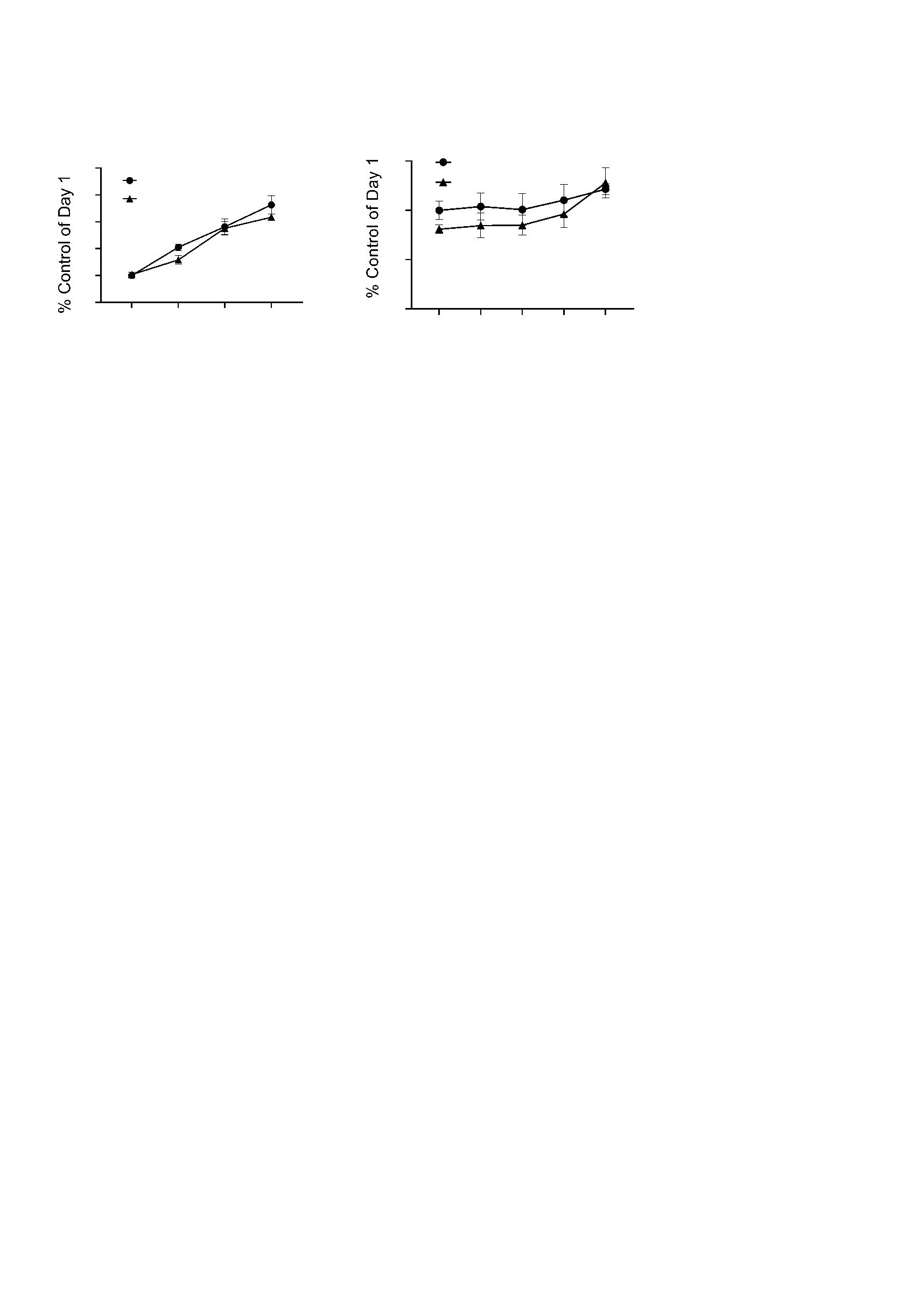


**Supplemental Figure 4**

**A**

2

1.6

1.2

0.8

0.4

0

*DRAM*

2

1.5

1

*IL-18*

**

** **

0.5

0

**

**

1.2

*MOAP1*

1

0.8

0.6

0.4

0.2

0

**

**

Control

E5

**B**

*IL-18*

*DRAM*

1.2

1.2

1

0.8

0.6

0.4

0.2

0

1

0.8

0.6

0.4

0.2

0

**

**

**

**

**

24h

48h

96h

24h

48h

96h

**C**

1.2

1

*MOAP1*

80%

60%

40%

20%

0%

0.8

0.6

0.4

0.2

0

**

**

24h

48h

96h

24h

96h


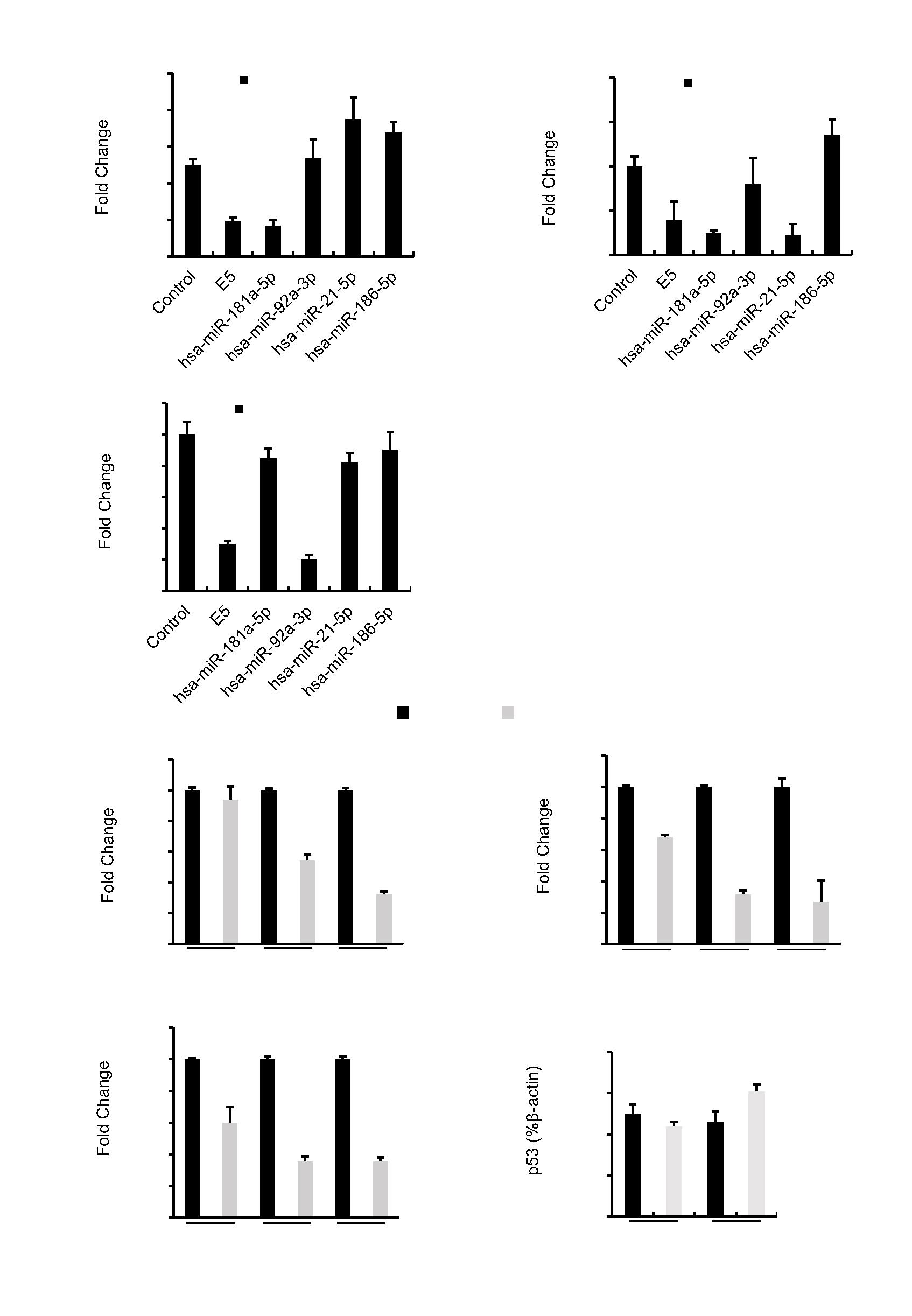


**Supplemental Figure 5**


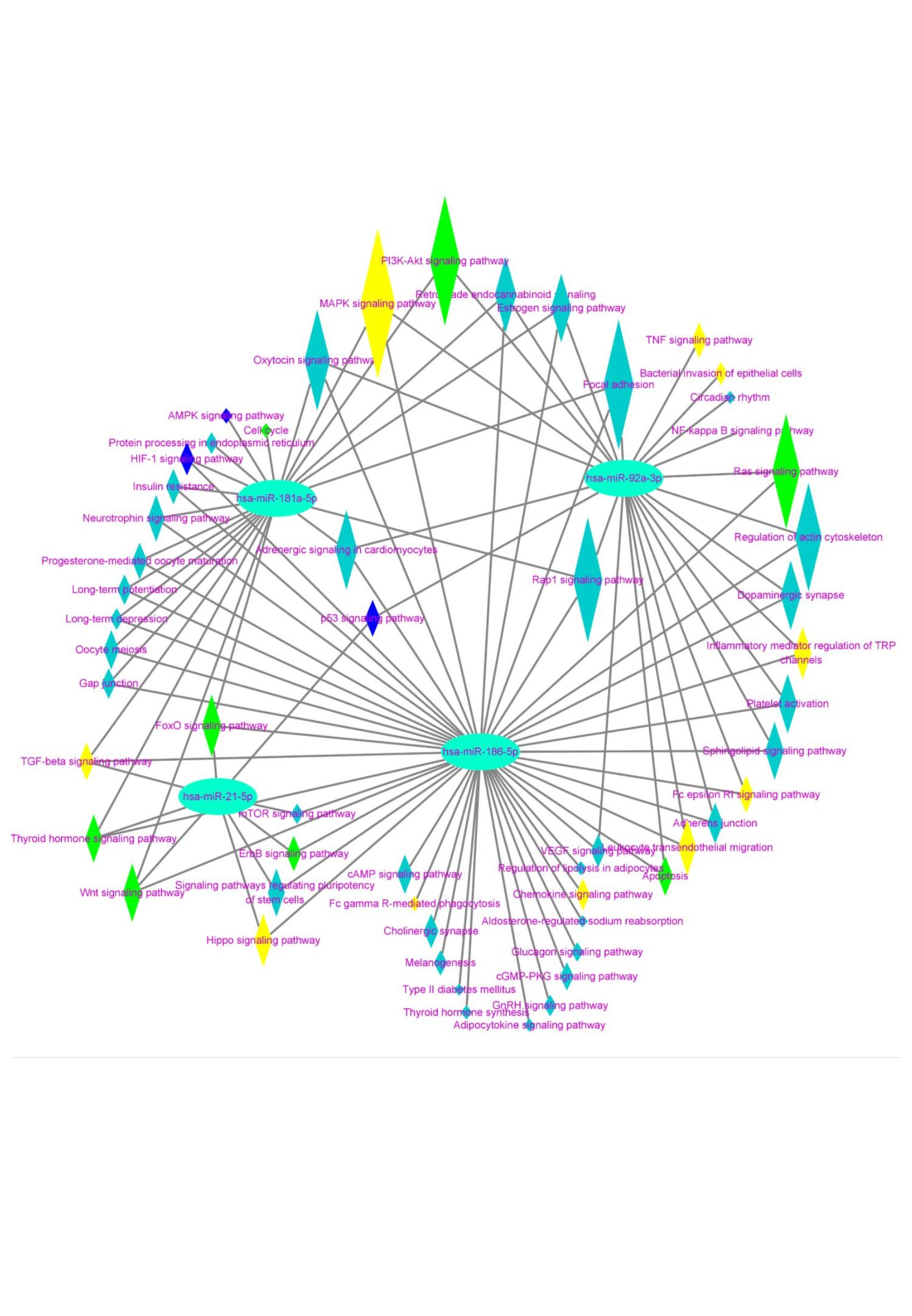


**Supplemental Figure 6**

**A**

Control

E5

**B**

Kidney

*p*=0.036

*p16*

*p*=0.253

*p21*

*p*=0.168

*IL-1β*

2.0

1.5

1.0

0.5

0.0

1.5

1.0

0.5

0.0

2.0

1.5

1.0

0.5

0.0

l

o

l

o

l

5

5

5

o

r

t

E

r

t

E

r

E

t

n

n

n

o

o

o

C

C

C

*p*=0.025

*p*=0.924

*IL-6*

*Mcp-1*

1.5

1.0

0.5

0.0

2.0

1.5

1.0

0.5

0.0

l

o

l

o

5

5

r

t

E

r

t

E

n

n

o

o

C

C


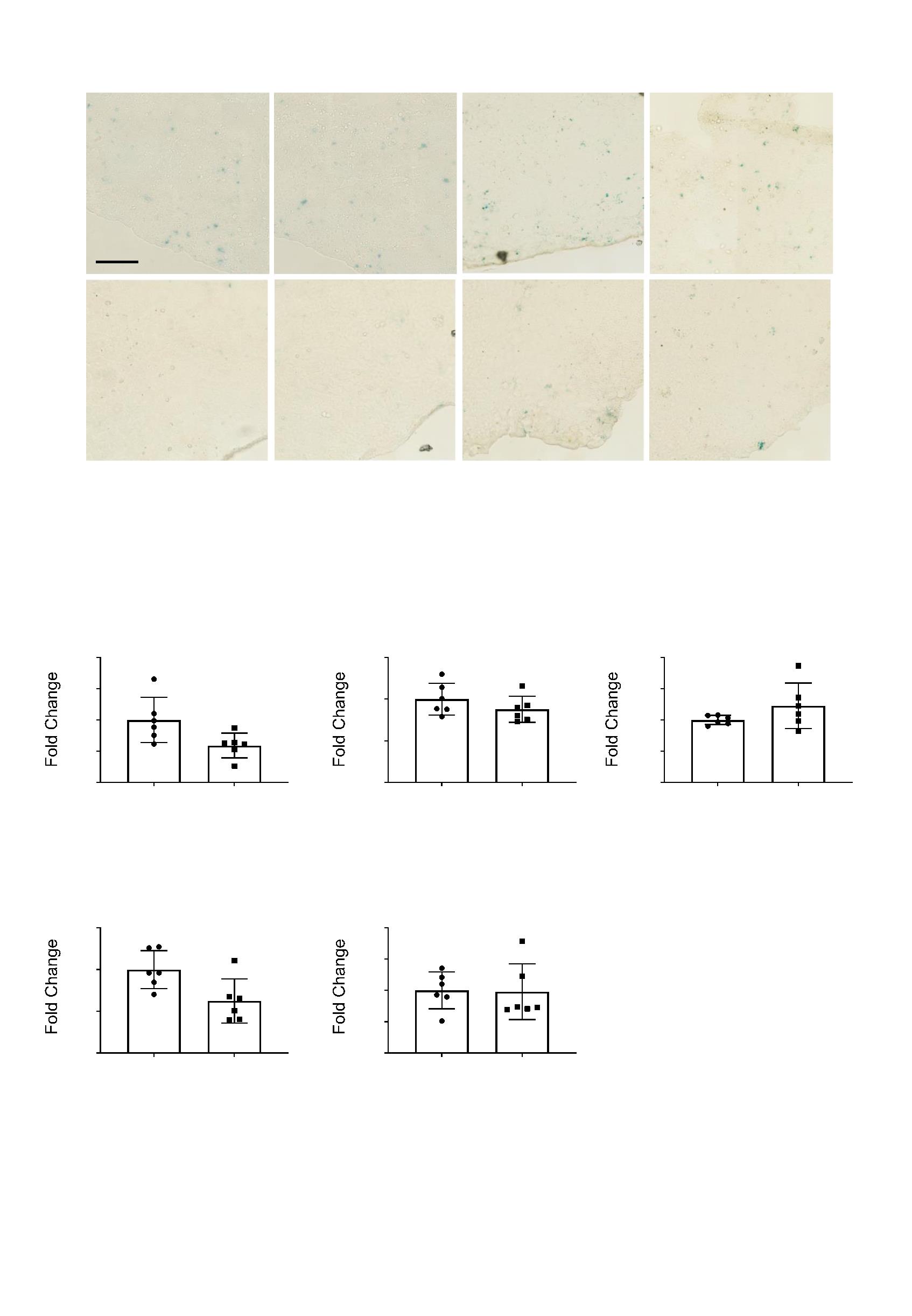


**Supplemental Figure 6**

Spleen

**C**

*p*=0.027

*p16*

*p*=0.419

*p21*

*p*=0.022

*IL-1β*

1.5

1.0

0.5

0.0

2.5

2.0

1.5

1.0

0.5

0.0

1.5

1.0

0.5

0.0

l

o

l

o

l

5

5

5

o

r

t

E

r

t

E

r

E

t

n

n

n

o

o

o

C

C

C

*p*=0.005

*IL-6*

*p*=0.549

*Mcp-1*

1.5

1.0

0.5

0.0

1.5

1.0

0.5

0.0

l

o

l

o

5

5

r

t

E

r

t

E

n

n

o

o

C

C

**D**

*p*=0.009

*p*=0.011

*p*=0.0006

miR-181a-5p

miR-92a-3p

miR-21-5p

80

60

40

20

0

15

15

10

5

10

5

0

0

l

l

l

5

5

5

o

o

o

r

E

r

E

r

E

t

t

t

n

n

n

o

o

o

C

C

C

*p*=0.009

miR-186-5p

100

80

60

40

20

0

l

5

o

r

E

t

n

o

C


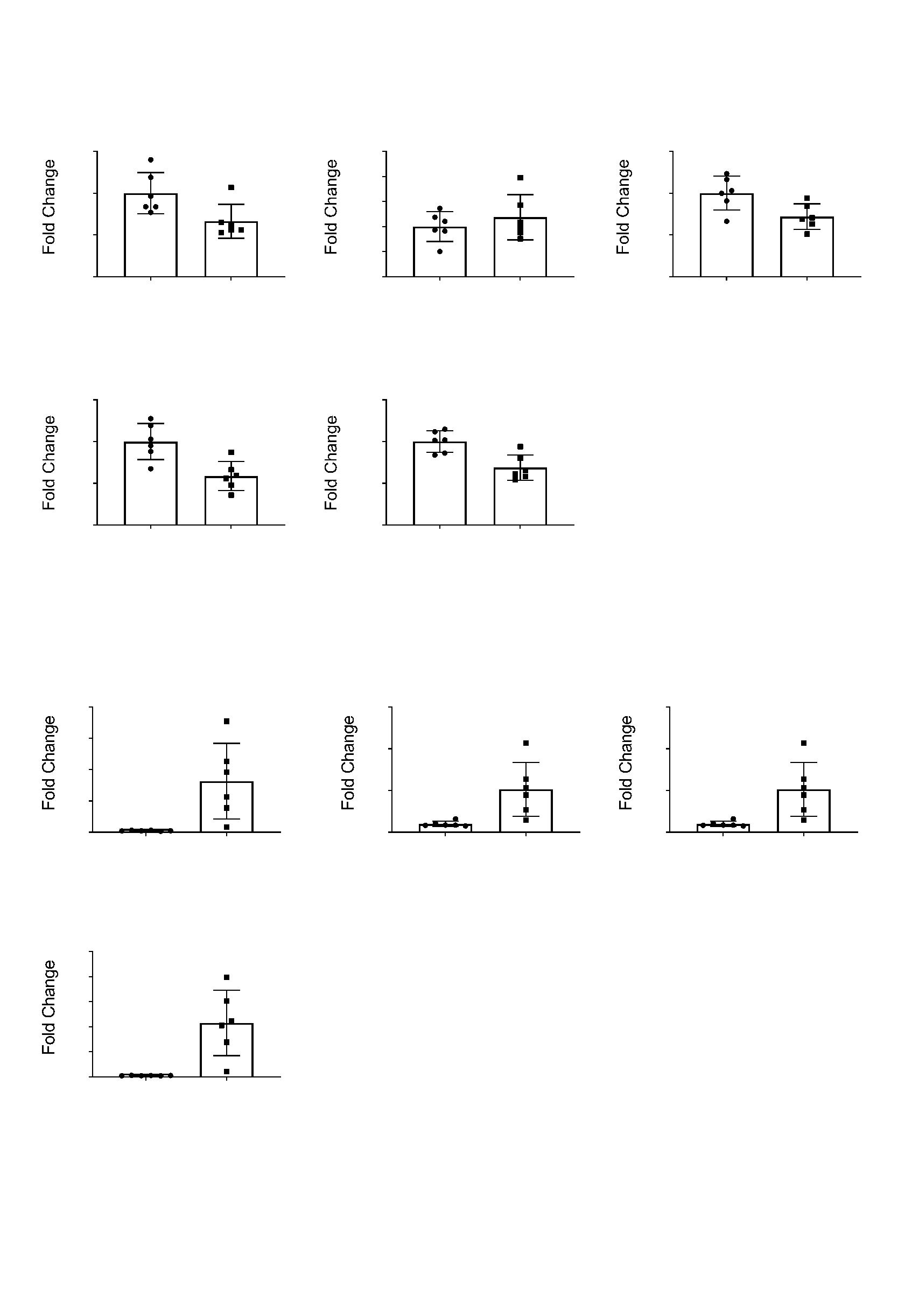

Supplement: Supplementary file 1 — Figure S1. Supplementary images of SA‐β‐gal staining of liver sections from Ercc1 −/Δ mice treated with AC83 EVs; scale bar is set to 2.5 mm. Figure S2. Comparative quantification of miRNA profiles derived from fibroblast EV, E69 EV, RP‐1 EV, and MSC EV. Figure S3. (A) qPCR assessment of miR‐181a‐5p, miR‐92a‐3p, miR‐186‐5p, and miR‐21‐5p expression levels at 96 h following transfection with 50 nM of each corresponding miRNA mimic in senescent IMR90 cells. (B–D) Additional evaluations of different combinations of miRNAs in senescent IMR90 cells. (E) Caspase 3/7 activity in nonsenescent and senescent IMR90 cells treated with E5 at 24, 48, and 96 h. (F) Relative viability of nonsenescent and senescent IMR90 cells treated with E5 at 24, 48, and 96 h. (G) Growth curves of non‐senescent cells treated with the E5 cocktail. Cell growth was monitored over 6 days posttreatment, and results are expressed as the fold change relative to Day 1. (H) Growth curves of senescent cells treated with the E5 cocktail. Cell growth was monitored over 16 days of posttreatment, and results are expressed as the fold change relative to Day 1. Data are shown as the mean ± SEM of three independent experiments. Figure S4. (A) Quantitative analysis of the relative expression levels of DRAM, IL‐18, and MOAP1 as determined by qPCR. (B) Time‐course qPCR analysis detailing the expression patterns of DRAM, IL‐18, and MOAP1 at 24, 48, and 96 h after transfection with the E5 miRNA cocktail. (C) The densitometer quantification of p53 at 24 and 96 h posttransfection with E5. Data are shown as the mean ± SEM. **p < 0.01. Figure S5. (A) Enrichment analysis of pathways regulated by each miRNA using DAVID based on their target genes. None‐age and disease related pathways were removed. The size of the diamonds indicates the target genes number. Yellow diamonds represent inflammation‐related pathways, blue for stress response‐related pathways, and green signifies pathways related to cell proliferation, survival, [file ACEL-24-e70071-s001.docx]
